# Tourette Syndrome Risk Genes Regulate Mitochondrial Dynamics, Structure, and Function

**DOI:** 10.3389/fpsyt.2020.556803

**Published:** 2021-03-10

**Authors:** Raymond A. Clarke, Teri M. Furlong, Valsamma Eapen

**Affiliations:** ^1^School of Psychiatry, University of New South Wales, Sydney, NSW, Australia; ^2^Ingham Institute for Applied Medical Research, Liverpool, NSW, Australia; ^3^School of Medical Sciences, University of New South Wales, Sydney, NSW, Australia; ^4^South West Sydney Local Health District, Liverpool Hospital, Liverpool, NSW, Australia

**Keywords:** Tourette syndrome genes, Tourette syndrome cause, Tourette syndrome etiology, mitochondrial fission, mitochondrial supply

## Abstract

Gilles de la Tourette syndrome (GTS) is a neurodevelopmental disorder characterized by motor and vocal tics with an estimated prevalence of 1% in children and adolescents. GTS has high rates of inheritance with many rare mutations identified. Apart from the role of the neurexin trans-synaptic connexus (NTSC) little has been confirmed regarding the molecular basis of GTS. The NTSC pathway regulates neuronal circuitry development, synaptic connectivity and neurotransmission. In this study we integrate GTS mutations into mitochondrial pathways that also regulate neuronal circuitry development, synaptic connectivity and neurotransmission. Many deleterious mutations in GTS occur in genes with complementary and consecutive roles in mitochondrial dynamics, structure and function (MDSF) pathways. These genes include those involved in mitochondrial transport (*NDE1, DISC1, OPA1*), mitochondrial fusion (*OPA1*), fission (*ADCY2, DGKB, AMPK/PKA, RCAN1, PKC*), mitochondrial metabolic and bio-energetic optimization (*IMMP2L, MPV17, MRPL3, MRPL44*). This study is the first to develop and describe an integrated mitochondrial pathway in the pathogenesis of GTS. The evidence from this study and our earlier modeling of GTS molecular pathways provides compounding support for a GTS deficit in mitochondrial supply affecting neurotransmission.

## Introduction

Gilles de la Tourette Syndrome (GTS) is a neurodevelopmental disorder with an estimated prevalence of 1% in children and adolescents (1). Neuroanatomical evidence suggests that GTS pathology is related to abnormal brain development and the physiological involvement of the cortico–striato–thalamo– cortical (CSTC) circuitry connecting the cortex, basal ganglia and thalamus (1). Clinical evidence further suggests the involvement of neurotransmitters such as dopamine, glutamate and γ-aminobutyric acid (GABA) (1). Epidemiological, phenomenological and genetic evidence demonstrate broad overlap between GTS and autism spectrum disorder (ASD) (2, 3) with both exhibiting high incidence in first-degree relatives, high monozygotic to dizygotic concordance (4), and with both conditions beginning during childhood with a high male preponderance. Furthermore, GTS and ASD share associated clinical features of compulsive behaviors, obsessions, involuntary movements (tics in GTS and stereotypies in ASD), poor speech control and echolalia common in both conditions (5). Attention deficit hyperactivity disorder (ADHD) is also present in both ASD and GTS (5, 6). GTS is over represented in ASD, with 5% having GTS and up to 40% experiencing tics (5). Similarly, the rate of autism in GTS exceeds that expected by chance, with reports of ASD in around 22.8% of children and 8.7% in adults (7), subclinical autistic symptoms occurring in a third of GTS populations, and a further two-thirds showing social deficits relating to the autism spectrum (8). Pharmaco-therapeutic agents such as the α2-adrenergic agonists Clonidine and Guanfacine and the antipsychotics such as Risperidone and Aripiprazole are usually the first-line of therapy for moderate to severe GTS. However, side effects are particularly problematic during childhood years when the symptoms are most predominant and often affect compliance and hence there is a critical need for targeted therapeutic development based on a better understanding of the genetic etiology of the disorder.

**Figure 1:**
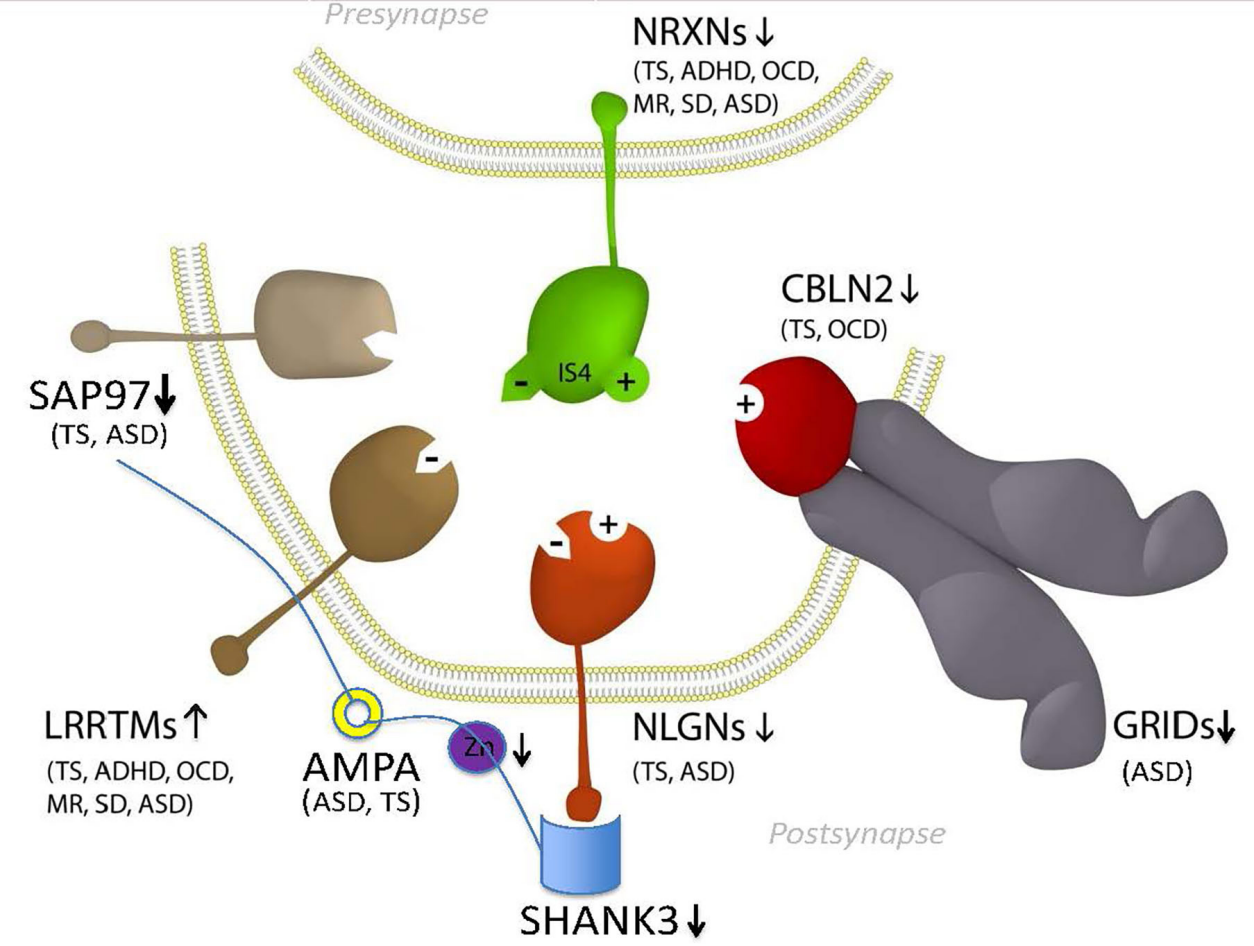
Model NTSC pathway to GTS (2)—implicates the full complement of known neurexins (NRXNs) and their trans-synaptic cell-adhesion ligand gene families through multiple means of enquiry: neuroligins (NLGNs); leucine-rich repeat transmembrane proteins (LRRTMs); cerebellin precursors (CBLNs), Glutamate ionotropic receptor delta type subunit (GRIDS) and the synapse associated proteins (SAPs/DLGs) (2, 11, 12) in addition to the ProSAP proteins that interact in the NTSC postsynaptic signaling pathway (2, 11, 13–24). Presynaptic NRXNs 1-3 form competitive trans-synaptic complexes with postsynaptic ligands NLGNs, LRRTMs and CBLN-GRID complexes in the formation and/or maintenance of neuronal circuitry within the brain. Vertical arrows indicate putative pathogenic dose effects. NRXN1 isoforms with (+) and without (–) the 30 amino-acid insert at splice site 4 (IS4) indicate competitive binding of NRXN1 between its ligands. NRXN4/CNTNAP2 binds across the synapse with SAP97/DLG1 (12, 25). Comorbidities listed are those associated with the gene mutations and copy number variations (CNVs) in ASD and GTS (2, 11, 12, 26–32). Zinc represents one environmental factor important for mitochondrial protein import/processing and function that is also known to affect SHANK3 function (23, 24). Moreover the Zinc transporter ZnT7 has been recurrently linked with GTS in GWAS (2).

GTS is one of the most heritable neuropsychiatric disorders of non-Mendelian inheritance, however, with the exception of the neurexin trans-synaptic connexus (NTSC) little is known regarding the molecular basis of GTS (9). One of the strongest mutation associations to date has been with *neurexin 1* and the genes encoding the NTSC which regulate neuronal circuitry development, synaptic connectivity and neurotransmission (2, 3, 10, 11). Members of the NTSC family of synaptic proteins bind across the synapse in different combinations to facilitate trans-synaptic cell-adhesion that helps establish and maintain neural circuits and neurotransmission within the brain. All major gene families of the NTSC (Figure 1) have been repeatedly mutated or otherwise associated with GTS and ASD (2, 11). Moreover, the number of mutations identified in and associated with the NTSC has continued to grow to such an extent that the NTSC now represents a collective mutation hot spot for GTS and ASD (2, 11, 13–22). Moreover, the NTSC model for GTS (Figure 1) provides a reliable starting point for further mutation pathway analysis into the mitochondrial regulation of neuronal circuitry development, synaptic connectivity and neurotransmission as it relates to mutations in GTS.

Neuronal Mitochondria: Up to 20% of the total energy consumed by humans at rest is attributable to brain activity despite a brain-to-body mass ratio of only 2% (33, 34). This high energy consumption by the human brain is largely attributed to requirements for synaptic transmission (33, 34). Neurons require particularly large amounts of energy for synaptic vesicle release and to power the ion pumps that restore ion gradients in the synapse following the ion influx associated with neuronal firing (34). These high energy demands are largely met by neuronal mitochondria, which also power other important neurodevelopmental processes including neurite outgrowth (35–40) which ultimately provides for optimal synaptic connections and neurotransmission. Mitochondria have additional roles in the neuron including calcium buffering, which is of particular importance in mitochondrial dynamics and neurotransmission (40). Although mitochondria are essential in almost every cell type, the extended branching structure and specialized function of neurons comes with unique demands over extended distances that render neurons especially sensitive to deficits in mitochondrial dynamics, structure and function. The sensitivity of neuronal development and function to mitochondrial deficiencies is corroborated by the strong association between the mutation of mitochondrial component molecules and neurological disorders (41), and there is increasing evidence that mitochondrial dynamics and dysfunction contribute to neuropsychiatric disorders including Schizophrenia (SCZ) and Bipolar Disorder (42–44).

The higher brain functions affected in neurodevelopmental and psychiatric disorders are thought to require precise spatiotemporal regulation of neuronal circuitry development. In this developmental process the relationship between neuronal outgrowth, synaptogenesis and synaptic transmission is widely appreciated. However, the requirements that these neuro developmental processes have on mitochondria is still emerging. In our pathway analysis, we outline the importance of mitochondrial dynamics, structure and function to neuronal outgrowth and development, synaptogenesis and neurotransmission as the basis for understanding the genetic etiology of GTS.

## Methods

**Figure 2:**
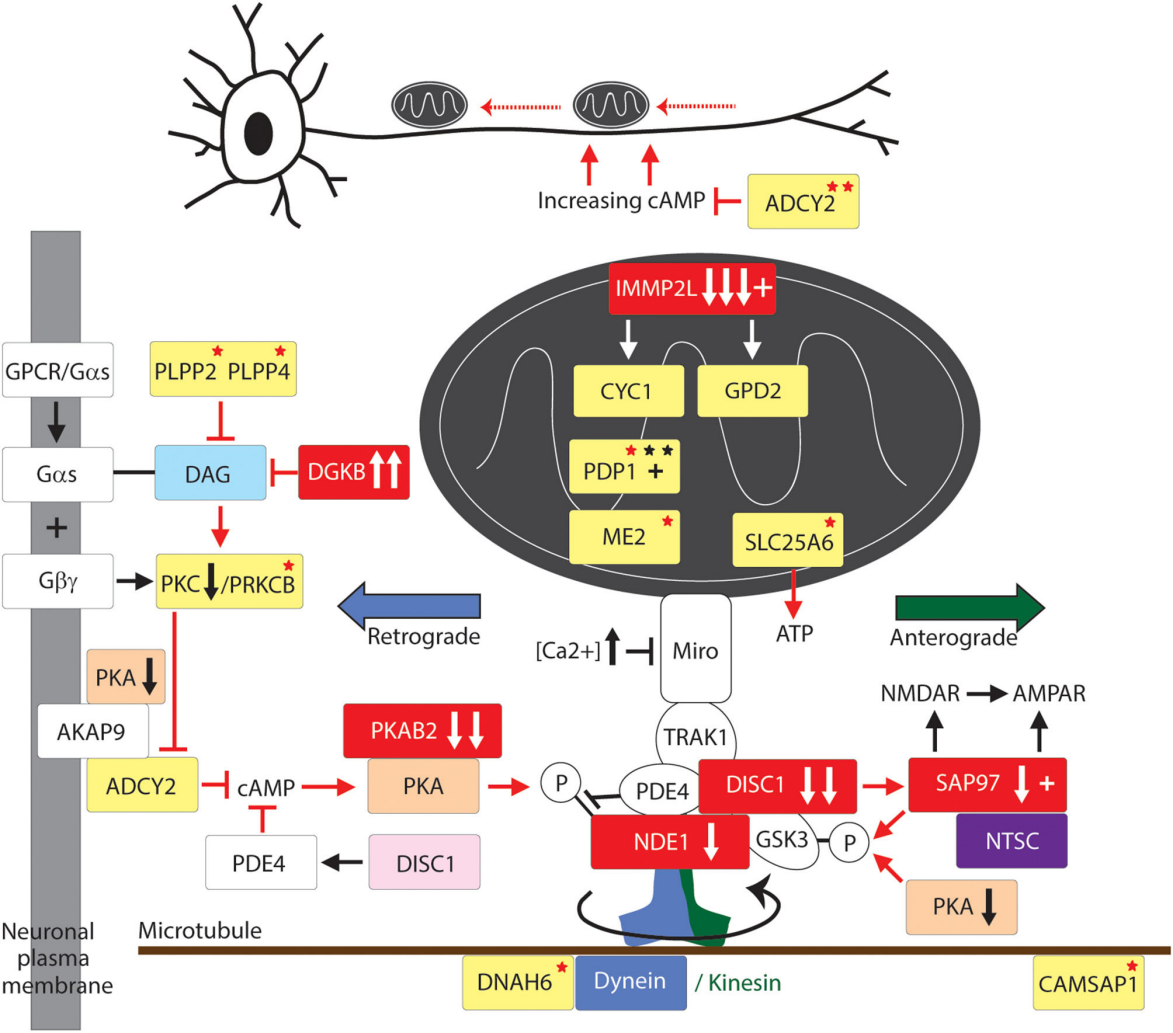
Mitochondrial transport pathway to GTS. Red fill indicates CNV deletion (arrows down) or duplication (arrows up); Yellow fill indicates GTS associated gene; Red asterisk^*^ indicates deleterious mutation; Black asterisk indicates another GTS genetic association; + indicates linkage; Red arrow indicates GTS affected pathway. Vertical arrows beside PKA and PKC indicate putative pathogenic dose effects.

**Figure 3:**
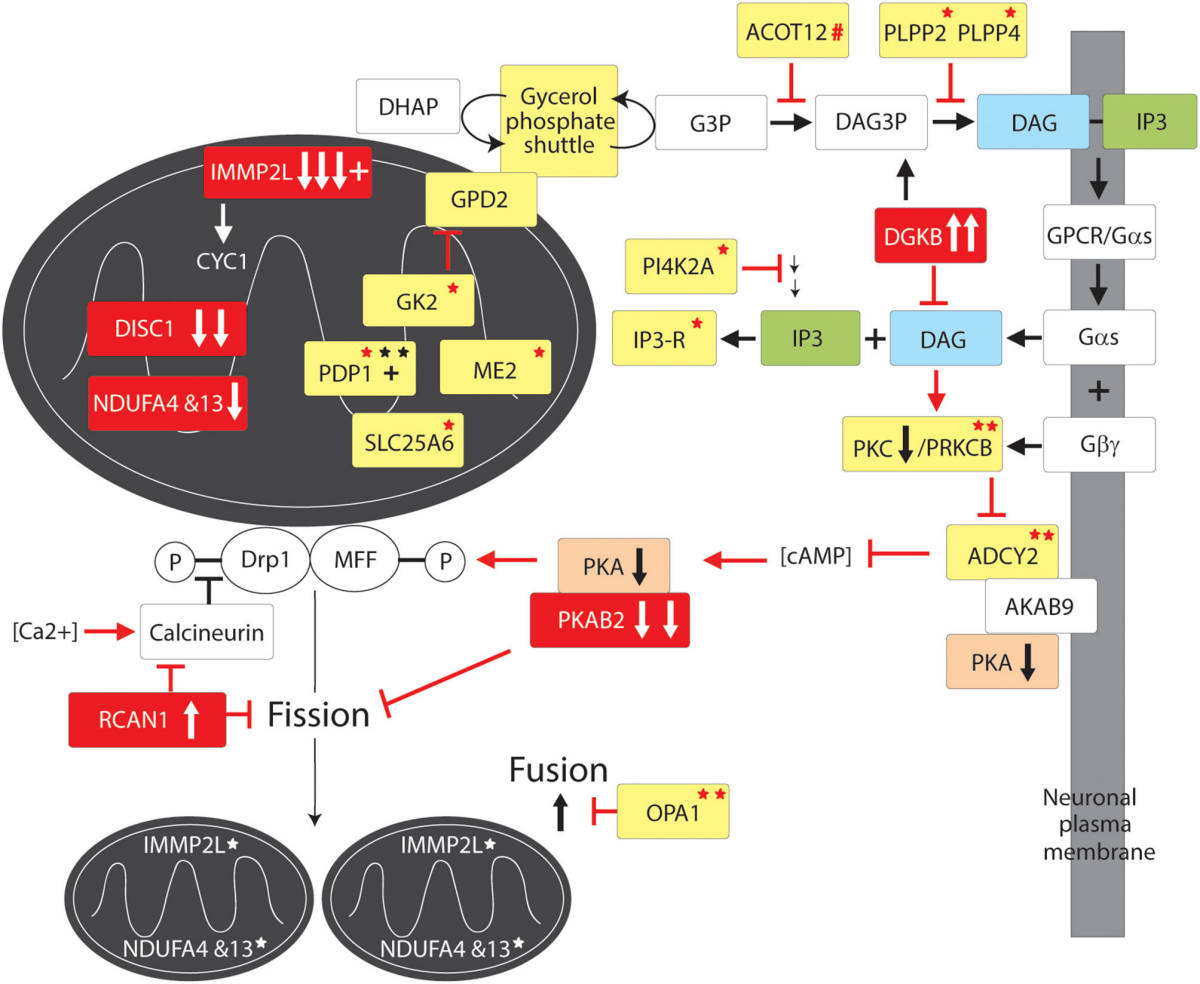
Mitochondrial fission and fusion pathway to GTS. Red fill indicates CNV deletion (arrows down) or duplication (arrows up); All other vertical arrows within boxes indicate putative pathogenic dose effects.Yellow fill indicates GTS associated gene; Red asterisk^*^ indicates deleterious mutation; Black asterisk indicates another GTS genetic association; # indicates adjacent to deletion or duplication; + indicates linkage; Red arrow indicates GTS affected pathway. Vertical arrows indicate putative pathogenic dose effects.

**Figure 4:**
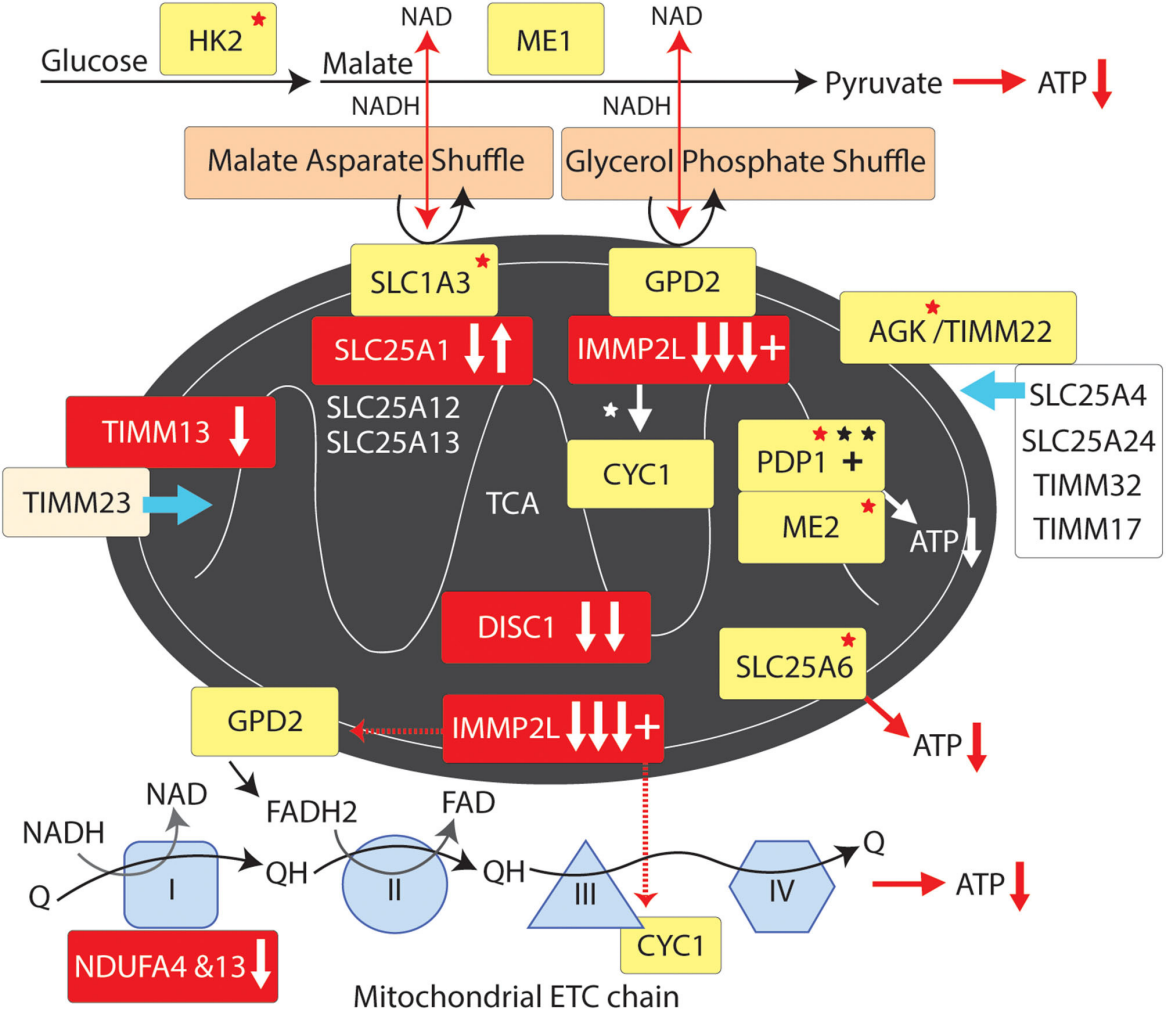
Mitochondrial supply pathway to GTS. Red fill indicates CNV deletion (arrows down) or duplication (arrows up); Yellow fill indicates GTS associated gene; Red asterisk^*^ indicates deleterious mutation; Black asterisk indicates another GTS genetic association; + indicates linkage; Red arrow indicates GTS affected pathway.

Data Mining and Mutation Pathway Analysis: In this study we integrated the findings from published and unpublished (database) sources including ASD brain gene expression profiling, GTS risk-gene mouse modeling of behavior, genome wide linkage studies, genome wide association studies, chromosomal translocations and copy number variations (CNVs), gene set analysis, haplotype sharing, cell modeling, whole exome sequencing (WES), and whole genome sequencing (WGS) of rare and common deleterious mutations in GTS and ASD (see sources in Table 1). In the case where a CNV spanned multiple genes only one gene was selected for inclusion in our pathway analysis (Table 2). In these cases, first priority was given to genes that directly regulate those pathways already implicated in GTS (9) including neuronal development (e.g., *SLIT2*), synaptic connectivity (e.g., NTSC pathway genes), synaptic function (e.g., *SAP97*) and neurotransmission (Figure 1) (9). Only then was the second priority exercised to assign a pathway to those genes that encode mitochondrial proteins. Following this selection process all mutations, deletions and duplications of mitochondrial protein genes in GTS were included within the mutation pathway analysis thereby eliminating any bias or cherry picking in our development of the first contiguous mitochondrial pathways to GTS (Figures 2–4).

**Table 1:** Data sources.

**Data type**	**Author**	**References**
Genome wide linkage studies	Curtis 2004	(45)
	Zhang 2002	(46)
	Verkerk 2006	(12)
	Simonic 1998	(47)
	IMGSAC 1998	(48)
	Barret 1999	(49)
	Shao 2002	(50)
	Shellenberg 2006	(51)
	Maestrini 2010	(52)
	TSAIC 2007	(53)
Genome wide association studies (GWAS)	Suarez-Rama 2015	(54)
	Lintas 2009	(55)
	Philippi 2005	(56)
	Eicher 2015	(57)
Copy number variations (CNVs)	Wang 2018	(58)
	Lintas 2017	(20)
	Malhotra 2012	(59)
	Johnstone 2015	(60)
	Fernandez 2012	(61)
	McGrath 2014	(62)
	Sundaram 2010	(63)
	Clarke 2018	(11)
	Bertelsen 2014	(64)
	Elia 2010	(65)
	Jang 2019	(66)
	Huang 2017	(31)
Whole exome sequencing (WES)	Wang 2018	(58)
	Sundaram 2011	(67)
	Gauthier 2011	(30)
Whole genome sequencing (WGS)	RK CY 2017	(18)
	Turner 2016	(21)
	Leblond 2019	(22)
Gene set analysis	Wittkowski 2014	(68)
	Clarke 2012	(2)
	Wang 2011	(69)
	De Leeuw 2015	(70)
Haplotype sharing	Casey 2012	(71)
Karyotype, LOH Analysis and PCR	Clarke 2018	(11)
	Boghosian-Sell 1996	(72)
	Petek 2001	(73)
	Zhang 2015	(74)
	Patel 2011	(75)
	Robertson 2006	(76)
	Clarke 2009	(25)
	Fang 2017	(11)
ASD brain gene expression profiling	Tang 2013	(77)
	Anitha 2013	(78)
	Anitha 2012	(79)
	Voineagu 2012	(80)
	Schwede 2018	(81)
	Ji L 2012	(82)
	Lintas 2009	(55)
GTS gene mouse modeling and behavior	Shen 2019	(83)
	Lu B 2008	(84)
	Kreilaus 2019	(85)
	Shoen 2019	(23)
Cell modeling	Lam 2019	(27)

**Table 2:** GTS risk genes in mitochondrial dynamics, structure, and function.

**Deleterious mutations**	
OPA1 (x2), MPV17^*^, PDP1^***^, ME2, SLC1A3/EAAT1/GLAST, MRPL44 (x3), MRPL48, MRPL3 - Familial, PTCD3, GK2, DPP4, SLC25A26, SLC25A6, SLC52A2, ATP5B, AGK/TIMM22, ACOX3, DGAT2, UBE3A, BCKDHA, ENOSF1, ACOT12	
**CNV duplications**	
*SLC25A1(x3), SAP97- Familial duplicated mediated downregulation (14)*	
**CNV deletions**	
*IMMP2L (x12) (x9 in ASD), IMMP1L (x3 in ASD), GPD2 (x1 in ASD), RMRP, SLC25A1 and Txrnd2 (x3), TIMM13, NDUFA4 (Familial deletion), NDUFA13/ETC Complex I, SAP97—Duplication mediated downregulation* (14)	
**Adjacent to deletion**	
*ACOT12*	
**Adjacent to duplication**	
*MGME1* mitochondria DNA maintenance	
^*^GTS linkage/association studies	
*MPV17*	Mutated and non parametric linkage analysis
*PDP1*	Mutated and Linked and Associated
*SLC25A4*	4q35Linkage region in Sib pairs
*NDUFS3*	D11S1377 in Africana families
*SAP97*	Parametric linkage in large Dutch pedigree
*IMMP2L*	Autism linkage
**Mitochondrial transport protein genes**	
*NDE1* (x2 deletions), *DISC1* (x3 deletions), *DNAH6, DNAH5,* * and DYNC2H1, KIF6 and KLC2* have been mutated, *KIF7* deleted and *KIF16B* is adjacent to a GTS deletion breakpoint * at 20p12.1*	
**Mitochondrial dynamics regulators**	
*OPA1 (x2), PRKAB2* (x 2 deletions + adjacent to deletion), *ADCY2 (x2), RCAN1 (duplicated), DGKB (x2 duplications), PLPP2 and PLPP4, PI4K2A and ITPR3* all mutated	
**Microtubule associated genes**	
*TUBB2A and TUBB2B* tubulin genes (duplicated), *TTLL1, TTLL2 and TTLL5* tubulin ligase genes mutated, *MICAL2 and MICAL3* microtubule regulators mutated, *KANK1* microtubule polymerization (x2 duplications), *CCT6A, BRPF1, SKA2, SPAST, KATNAL2, MARK2, TUBGCP5* and *CAMSAP1* which regulates microtubule dynamics and neurite outgrowth and *CAMD1* involved in microtubule stability and radial neuronal cell migration in the developing cerebral cortex lies immediately adjacent to the deletion of the Titin gene in GTS	
**Ubiquitin ligase genes**	
*UBE3A (duplicated), UBE4A, DTX3, RNF41, RNF213 (x2), SH3RF3, SHPRH, WWP2, UBR4 and TRIM37 all mutated*	
**Ubiquitin modifying genes**	
*UBE4B* ubiquination factor, *USP1 and USP47 and USP34* ubiquitin peptidases, *CYLD and BIRC6* all mutated	
**Cellular energy metabolism**	
*HK2, ME1* (from GTS associated 33 metabolic enzyme gene set)	

## Results and Discussion

### Mitochondrial Dynamics: Mitochondrial Transport

To optimize the development and function of neuronal circuitry, mitochondria need to be located at the right place at the right time in sufficient numbers and to be functioning at optimum efficiency. Given that neurons are often hyper-extended with a complex network of axonal and dendritic branches, such optimisation requires the active bidirectional transport of mitochondria to their required destinations along microtubule tracks (39, 86). This may involve long-distance transport of mitochondria from the soma, where the majority of mitochondrial biogenesis occurs, to the pre- and post-synaptic termini where demands for mitochondrial homeostasis is highest (39, 86). Mitochondrial transport is also thought to optimize mitochondrial fission, fusion and function (39, 86).

### Dynein and Kinesin Motor Proteins

Transport of mitochondria is regulated by the dynein and kinesin motor proteins through their interaction with the microtubule cytoskeleton of the cell (Figure 2). The kinesins (KIFs) mediate anterograde mitochondrial transport away from the soma of the neuron while dynein complexes regulate retrograde transport (87). *DNAH6, the dynein axonemal heavy chain* 6 gene implicated in mitochondrial depletion syndrome is mutated in GTS as are *DNAH5* and a number of kinesin genes including *KIF6* and *KIF7* (Figure 2) (58). Moreover, *DYNC1H1* which encodes the dynein 1 heavy chain mitochondrial transporter is mutated in ASD and *KIF1A* which encodes the Kinesin 1A mitochondrial transporter is disrupted and duplicated in ASD (18, 20).

KIFs form homo and heteromeric complexes in the regulation of organelle transport including but not limited to mitochondria (88). The importance of the motor proteins, and the microtubule network they travel on, in nervous system development and function is evidenced from their strong association with neurological phenotypes. Mutations in KIF5-family members give rise to a range of dominant negative phenotypes including deficits in mitochondrial transport, structure and function and reduced activity of the electron transport chain (ETC) (89, 90), axonal degeneration and aberrant synaptic transmission (Figure 2) (58, 91). GTS mutations have also been identified in genes that regulate tubulin and microtubule dynamics including: duplication of the *TUBB2A* and *TUBB2B* tubulin genes and the mutation of *TUBB3* (58); mutation of the *TTLL1, TTLL2*, and *TTLL5* tubulin ligase genes (58); recurrent duplication of the microtubule polymerization gene *KANK1* implicated in spastic paraplegia (58, 92); and mutation of the *CAMSAP1* gene that regulates microtubule dynamics and neurite outgrowth (Table 2 and Figure 2) (58, 93).

### Mitochondrial Transport Adaptor and Accessory Proteins

Mitochondrial transport provides for the site-specific requirements and function of the neuron (39, 86, 94–97) and it has been demonstrated that mitochondria directly regulate synaptic transmission (94). Furthermore, synapses with mitochondria can sustain repeated cycles of neurotransmitter release whereas the transport of mitochondria either in or out of the synapse dynamically modulates this synaptic strength (95–97). To halt the transport of mitochondria at a required destination such as the synapse requires a braking system. In this respect, synaptic firing renders synapses to be sites of high calcium influx. After synaptic firing the high (Ca^2+^) acts to halt mitochondrial transport through the action of the Ca^2+^ sensitive GTPase of MIRO which is embedded within the outer mitochondrial membrane which then inactivates the molecular motor kinesin (Figure 2). To summarize, the precise location and relocation of mitochondria closely matches the site-specific requirements of the neuron including a rich supply of energy, in the form of ATP, to power the synaptic calcium ion pumps that expel calcium from the cell and for direct mitochondrial buffering of Ca^2+^ (98–100). Furthermore, mitochondria directly regulate the strength of synaptic transmission (94).

Motor proteins interact with mitochondria through adaptor and accessory proteins (98, 101) that determine the direction of mitochondrial transport (Figure 2). The mitochondrial transport adaptor proteins TRAK1 and TRAK2 link mitochondria to the motor proteins kinesin and dynein (102). TRAK1 and TRAK2 interact with the mitochondria through the Ca^2+^ sensitive GTPases MIRO1 and MIRO2 embedded within the outer mitochondrial membrane (Figure 1) (102–106). MIRO and TRAK work in concert with the transport accessory proteins DISC1 and NDE1 that determine the direction of mitochondrial movement (Figure 2). While no mutations in *MIRO* or *TRAK* have been identified (107) both *DISC1* and *NDE1* display recurrent hemizygous deletion in GTS, ASD and SCZ (Table 2) (58–62). DISC1 is localized predominantly to mitochondria and in synapses, centrosomes, nuclei, endoplasmic reticulum and the Golgi (108, 109). DISC1 forms oligomers that interact with kinesin (110) and dynein (111), MIRO and TRAK, and the mitochondrial transport accessory proteins LIS1, GSK3β, NDE1 and its homolog NDEL1 (Figure 2) (112). DISC1 promotes anterograde and retrograde mitochondrial transport in a dose dependent manner in both axons and dendrites (112–114) possibly through blocking SNPH-mediated anchoring of mitochondria (115). DISC1 is also directly linked to anomalies in mitochondrial fission, fusion, structure and function (112–114).

NDE1 interacts with DISC1, TRAK1, NDEL1, LIS1, and with the dynein motor protein to promote retrograde axonal transport (Figure 2) (112). NDE1, and its close homolog NDEL1 form a complex with LIS1 that regulates neuronal proliferation, differentiation, and migration within the brain (116). NDE1 is a centrosomal protein with a crucial role in the growth of the cerebral cortex (116). Homozygous frame shift mutations in NDE1 are associated with extreme microlissencephaly (117, 118), whereas heterozygous deletions (LOH) in NDE1 are associated with GTS, ASD, and SCZ (62). Interestingly, the subcellular localization of NDE1, its protein-protein interactions and its regulation of retrograde mitochondrial transport, are all modulated through its phosphorylation by AMP-activated protein kinase A (AMPK/PKA) (Figure 2) (119). This regulatory association between NDE1 and PKA is note-worthy on many counts. Firstly, the gene encoding beta subunit 2 of PKA (PRKAB2) is recurrently deleted in GTS (Table 2) (58, 63, 120, 121) and PKA activity is greatly decreased in the frontal cortex of subjects with regressive autism (122). Secondly, cAMP mediated PKA phosphorylation of NDE1 at threonine residue 131 regulates NDE1's all-important interactions with NDEL1 and LIS1 that are thought to activate dynein and facilitate its ability to move high-load cargo like mitochondria (123, 124). Thirdly, PKA's activation by rising cAMP levels provides a mitochondrial transport switch that can be activated on depletion of cellular ATP. Finally, DISC1 modulates the phosphorylation of NDE1 by PKA through its regulation of PDE4, a cAMP-hydrolyzing enzyme which creates a co-complex with DISC1 and NDE1 (Figure 2) and LIS1 and NDEL1 (112, 119, 125).

Mitochondria also localize to sites of neuronal branching. During development of neuronal circuitry the axons are guided to their target sites by extracellular guidance molecules like DSCAM, ROBO1, SLIT2 and SLIT3. *SLIT3* has been recurrently associated with GTS (2, 45, 46) as has *SLIT2* with ASD (2, 68, 69). Moreover, *DSCAM* and *ROBO1* have been recurrently mutated in ASD as have *DSCAM*'*s* DNA regulatory elements (21, 22).

Neuronal growth cone navigation also relies on intracellular changes to microtubule and F-actin architecture downstream of these guidance cues (126), for example CAMSAP1 which was mentioned above in relation to its regulation of microtubule dynamics and neurite outgrowth (Table 2 and Figure 2) (58, 93). Furthermore, AMPK/PKA regulates F-actin cytoskeletal dynamics (127). After extension to their target sites axons undergo local branching to establish the appropriate functional connections between pre- and postsynaptic neuronal termini. The intracellular mechanism that regulates this axonal branching also involves PKA through its regulation of mitochondrial transport and recruitment to sites of future axon branching (128). Here, neuronal depolarization-induced rebalance of mitochondrial motility between anterograde and retrograde transport underlies the formation of axonal branches (128). Axon branching is formed in an ATP-depletion dependent manner through an increase in activated/phosphorylated PKA—a function which can be recapitulated by the pharmacological activation of PKA (128). Following neuronal depolarization there is an increase in anterograde transport of mitochondria into axons thus providing a mechanism for mitochondrial relocation and recruitment to sites of high energy demand that would appear to include sites of future branching. Moreover, the continued localization of mitochondria at branch points correlates with the longevity of axonal branches indicating a probable role for mitochondrial localization in the maintenance of axon branches (128). To summarize, a role for mitochondria in neuronal function and neurological disease has been established. Moreover, the role of mitochondrial transport in GTS is greatly strengthened by the interacting roles of the dynein motor protein, DISC1, NDE1, and PKA in mitochondrial transport and the complementary nature of their deleterious mutations in GTS and ASD (Table 2) (61–63, 112).

The master regulator glycogen synthase kinase 3β (GSK3β) is phospho-deactivated by another master regulator AMPK/PKA (129–131). This is important given that GSK3β associates with both DISC1 and TRAK1 in the regulation of mitochondrial transport (Figure 2) (112). In the synapse GSK3β is also deactivated by SAP97 downstream of DISC1 (132) (Figure 2). *SAP97* is linked to GTS (12) and downregulated in GTS and ASD (11) and forms part of the high-risk NTSC pathway to GTS (Figure 1) (2, 12). As such, SAP97 functions at the intersection of the NTSC, DISC1 and mitochondrial transport pathways to GTS (Figures 1, 2) (2, 11, 12). GSK3β is also translocated into the mitochondria where it regulates mitochondrial homeostasis (112, 129, 133, 134). In the mitochondria GSK3β regulates the structure and function of the inner mitochondrial membrane (134). This is noteworthy given that the LOH and/or downregulation of *SAP97, DISC1*, and *PRKAB2* in GTS (2, 11, 12, 58, 61–63) are all consistent with stronger activation of GSK3β which is in turn consistent with the success of lithium chloride in the treatment of psychosis through its highly selective phospho-deactivation of GSK3 in both the cytosol and mitochondria (12, 134).

### Mitochondrial Fusion and Fission

The constant optimisation of mitochondrial function requires mitochondria to undergo fusion and fission. Fusion of suboptimal mitochondria with healthy mitochondria creates larger healthier mitochondria where the damage is diluted (98, 135). Fission of mitochondria can rapidly increase the number of healthy mitochondria to allow for their wider distribution in the extended network of neuronal branches and boutons (98, 135). Fission can also help separate out damaged mitochondrial components for clearance by mitophagy (98, 102, 135). Conversely, if transport of mitochondria is retarded or otherwise defective mitochondria are less likely to merge thereby decreasing the clearance of damaged mitochondria and the overall health of the mitochondrial pool (39, 86).

### Mitochondrial Fusion

Fusion of the outer mitochondrial membrane is coordinated by Mitofusins 1 and 2 (Mfn1/2) whereas fusion of the inner mitochondrial membrane is regulated by Optic Atrophy 1 (OPA1) (136, 137). This is most relevant as *OPA1* is recurrently mutated in GTS (Table 2 and Figure 3) (58) and the levels of MFN1, MFN2, and OPA1 are decreased in the temporal lobe of the autistic brain, and there are deficits in MFN1 and MFN2 in Fragile X syndrome (Figure 3) (58, 77, 83). Mfn1/2 and OPA1 act through the formation of complexes both within and across the membranes (136, 137). Mfn2 coding mutations appear to inhibit mitochondrial fusion by forming a complex, in a dominant-negative fashion, with wild-type Mfn1 and Mfn2 (138). Mutations in Mfn2 cause Charcot Marie Tooth Disease Type 2A, a severe and early onset motor and sensory peripheral neuropathy with autosomal dominant inheritance (111, 139). These Mfn2 mutations promote mitochondrial fragmentation in dorsal root ganglion neurons and impair axonal mitochondrial transport which is suggestive of a link between mitochondrial fission/fusion equilibrium and mitochondrial transport (140). This link is supported further by the physical interaction between Mfn2 and the MIRO complex (141) and the finding that Purkinje-neuron-specific deletion of Mfn2 in mice (total knockout of Mfn2 is embryonic lethal) impairs mitochondrial fusion and the dendritic localization of mitochondria, dendrite development, degeneration of Purkinje neurons (142, 143). Similar to the situation in mice, Mfn2 loss-of-function in zebrafish reduces mitochondrial transport and depletes mitochondria from distal axons (144). Acting in a similar dominant-negative fashion to Mfn2, hypomorphic mutations in OPA1 cause dominant optic atrophy (DOA), the most common cause of hereditary blindness. Dominant optic atrophy is characterized by the early loss of retinal ganglion cells and degeneration of the optic nerve (145). Moreover, DOA patients often present with neurological disorders, including ataxia, myopathy, deafness and peripheral neuropathy, indicating an essential neurological role for OPA1 (146, 147). Like Mfn2, knockout of Opa1 in mice is embryonic lethal (141) whereas Opa1 LOH in mice recapitulates the DOA seen in patients, including early-onset degeneration of the optic nerve and vision loss (148). *In vitro* experiments indicate that Opa1 has a critical role in dendritogenesis and synaptogenesis. Knock-down of Opa1 in cultured rat cortical neurons promotes mitochondrial fragmentation, decreases expression of ETC components, mitochondrial DNA content, dendritic outgrowth and synapse formation (149). As such, the characterization of mitochondrial phenotypes in those GTS patients identified with recurrent *OPA1* mutations is eagerly anticipated (Figure 3) (58).

### Mitochondrial Fission Regulatory Genes in GTS

The number and the complementary nature of the deleterious GTS mutations identified in mitochondrial fission pathways provide compelling evidence for mitochondrial fission deficiency in the etiology of GTS. The fission of the mitochondrial membranes is dependent on Dynamin-related protein1 (Drp1) (150–160) which is downregulated in the brain of patients with ASD (78–81). Drp1 also regulates peroxisomal fission and proliferation (161). Primarily a cytosolic enzyme, Drp1 translocates to the outer mitochondrial membrane when dephospho-activated by the Ca^2+^-activated phosphatase calcineurin/PPP3CA (Figure 3) (162, 163). Conversely, calcineurin is inhibited by RCAN1 which blocks Drp1 translocation to the mitochondria (164, 165) and hence it is of immense interest that *RCAN1* has been duplicated in GTS and *Calcineurin* is downregulated in the cerebral cortex of patients with ASD (Figure 3) (47, 63, 80, 81, 166). Calcinueurin activated Drp1 is then recruited to the outer mitochondrial membrane by phospho-activated mitochondrial fission factor (MFF). MFF is phospho-activated in neurones by PKA in response to increasing levels of cAMP (Figure 3) (167, 168). Activated Drp1 then assembles into spirals around the mitochondrion, which constrict and ultimately divide the organelle in two while dynamin-2 catalyzes the final membrane scission event (169). As mentioned earlier, the gene for beta subunit 2 of cAMP activated PKA (*PRKAB2*) is recurrently deleted in GTS (58, 63). Furthermore, cAMP levels in the brain are largely regulated by *ADCY2* (170), the adenylate cyclase gene recurrently mutated in GTS including the loss of an *ADCY2* intron/exon splice site in GTS (58). *ADCY2* is also mutated in bipolar disorder and a mutation in the ADCY2 binding site of the AKAP9 synaptic scaffolding protein has been found associated with SCZ (54, 170). This is fascinating since AKAP9 acts as a synaptic membrane anchor for ADCY2 and PKA which keeps this primary target of cAMP (PKA) in close proximity to the primary cAMP generator ADCY2 (170–173).

ADCY2 is activated by G-protein signaling through release of the alpha subunit of trimeric G protein (Gαs) (170, 174). Gαs is released when the G protein coupled receptor (GPCR) in the plasma membrane is bound by an extracellular regulatory molecule such as the neurotransmitter dopamine (Figure 3) (174, 175). When released, Gαs activates Phospholipase-C which in turn acts on phospholipid (phosphatidylinositol-triphosphate) within the plasma membrane cleaving off the inositol triphosphate (IP3) second messenger which frees yet another important second messenger diacylglycerol (DAG) (175). DAG activates Protein Kinase C (PKC), which in turn activates ADCY2 and other proteins (Figure 3) (175). Notably, the gene encoding PKC subunit B (PRKCB1) is mutated in GTS (Figure 3) (58) as well as being both linked and strongly associated with ASD, moreover, PKC activity is significantly reduced in the frontal cortex of subjects with regressive autism (55, 56, 82). *PRKCB1* is also associated with nominal autistic-like traits in the general population (176). Another compelling finding that links the PKCB/ADCY2 pathway to GTS is the recurrent duplication of the DAG kinase gene in GTS (DGKB) (58). DGKB terminates DAG-based signals by reducing DAG levels by converting DAG to diacylglycerol-3-phosphate (DAG3P) (Figure 3). Moreover, the phosphatases PLPP2 and PLPP4 which convert DAG3P back to DAG are both mutated in GTS (Figure 3) (58). It is also worthy of mention here that PI4K2A, an enzyme in the synthesis pathway of phosphatidylinositol-triphosphate with the potential to limit the bioavailability of both the IP3 and DAG second messengers, is mutated in GTS. This together with a GTS mutation in the IP3 receptor ITPR3 (Figure 3 and Table 2) (58) suggests the potential for an IP3 signaling affect in GTS notwithstanding ambiguity with regards to the calcium sensitivity of ACDY2 (170, 174, 175). To summarize, there is an impressive number of GTS gene mutations with the potential to limit the activation of Drp1, or the MFF-mediated recruitment of Drp1, for mitochondrial fission (Figure 3).

### Optimisation of Mitochondrial Supply

Many of the genes mutated in GTS regulate mitochondrial function. Most notable is the *IMMP2L* gene commonly disrupted/deleted in GTS (64, 72–75, 84). *IMMP2L* encodes a mitochondrial peptidase (inner mitochondrial membrane peptidase-2-like protein) which processes other mitochondrial proteins within the inner mitochondrial membrane (**IMM**) (Figure 4 and Table 2) (84). The *IMMP2L* association with GTS was first reported in a GTS family with a balanced *t* (7;18) (q22–q31; q22.3) translocation that disrupted the *IMMP2L* gene (72). More recently a Danish study reported 5′-end intragenic deletions in *IMMP2L* in seven out of a cohort of 188 GTS patients (3.7%) which was significantly higher than that of the control population (64). The IMMP2L gene has been repeatedly linked to ASD inheritance at the Autism 1 (AUTS1) locus (48–52). In addition, *IMMP2L* has demonstrated haplotype sharing in multiple ASD populations and deleterious exon deletions have been identified in ASD individuals and families (22, 52, 65, 66, 71, 74, 177) at significantly higher frequency than in control populations. Furthermore, reducing Immp2l dose in mice causes behavioral changes relevant to GTS behavioral deficits (85).

IMMP2L cleaves IMM signature signal peptides from a number of IMM proteins including cytochrome C1 (CYC1) and mitochondrial glycerol-3-phosphate dehydrogenase (GPD2) (84). CYC1 (oxidative phosphorylation complex 111 subunit 4) is a heme-containing subunit of the cytochrome complex of the electron transport chain (ETC). CYC1 has an important role in accepting electrons from the Rieske protein and transferring them to Cytochrome C in the respiratory chain. On the other hand GPD2 functions as part of the glycerol phosphate shuttle (Figure 4). GPD2, which is activated by IMMP2L, is located on the outer surface of the inner mitochondrial membrane where it catalyzes the interconversion of glycerol-3-phosphate (G3P) to dihydroxyacetone phosphate. Interestingly, mitochondrial glycerol kinase (GK2) which generates G3P is also mutated in GTS (58). Together, GPD1 and GPD2 constitute the glycerol phosphate shuttle, which generates FADH_2_ for the mitochondrial ETC and NAD+ for glycolysis in the cytosol. The coordinated action of GPD1 and GPD2 results in the transfer of two reducing equivalents from G3P to the mobile electron carrier ubiquinone (Coenzyme Q10) which in turn passes these electrons to CYC1 located downstream in the ETC (80, 81, 178–180).

A recurrent functional variant in the glutamate aspartate transporter GLAST/*SLC1A3* has been identified in GTS (181) (Table 2). SLC1A3 is also of interest as it imports glutamate from the cytosol into the mitochondrial matrix and exports aspartate from the matrix to the cytosol at varying levels in different cell types including astrocytes and neurons (178, 181). In addition to reducing glutamate signaling within the synapse the malate aspartate shuttle, like the glycerol phosphate shuttle, provides NADH to the ETC to generate ATP and NAD+ for another round of glycolysis (Figure 4) (178). In addition, the mitochondrial ADP/ATP exchange transporter SLC25A6 is mutated in GTS as is the mitochondrial ATPase ATP5B (Table 2) (58). SLC25A4 is a nuclear encoded protein located at the 4q35 GTS linkage locus (2, 46) (Table 1). SLC25A4 is transported into the mitochondria by the TIMM22 mitochondrial translocase complex inclusive of AGK, a vital component of TIMM22 involved in its assembly and function, and which is downregulated in the cerebral cortex of autism sufferers (80, 81). Importantly, *AGK* is mutated in GTS as is the *TIMM13* (Table 2 and Figure 4) (58, 182, 183). TIMM13 facilitates translocation of the TIMM23 translocase into the IMM which in turn forms a translocase complex with TIMM17A/B, which itself is translocated into the IMM by TIMM22 (184). Importantly, TIMM17A/B facilitates the translocation of two additional glutamate/aspartate exchange transporters into the IMM, namely SLC25A12 and SLC25A13 (Figure 4) with the former being downregulated in the brain of patients with ASD (78, 79).

### Mitochondrial Maintenance

The MPV17 channel protein (53, 58) that regulates the transmembrane potential of the IMM and mitochondrial DNA maintenance has been mutated in GTS (Table 2), moreover, the *MPV17* gene is located at the 2p23.2 non-parametric linkage locus identified in GTS (53) (Table 2). *MGME1* which also regulates mitochondrial DNA maintenance is located immediately adjacent to a genomic DNA duplication at 20p11 in GTS (Table 2) (58). *RMRP*, a gene which regulates mitochondrial RNA processing, is deleted in GTS as is the *PTCD3* gene which regulates translation in the mitochondria (Table 2). In addition, a number of mitochondrial ribosomal protein genes are mutated in GTS: *MRPL44* was found mutated in 3 unrelated GTS patients (Table 2) (58), a mutation in *MRPL3* was found segregating with GTS in a large affected family (67) and *MRPL48* was found mutated in another GTS patient (Table 2) (58). Together these findings are reminiscent of the *MRPL19* gene association with ASD, Dyslexia and Reading Disorder (57).

## Conclusion

The mitochondrial pathways involved in GTS overlap and interact making it possible to trace these pathways to common endpoints in mitochondrial dynamics and supply. While it is unlikely that all of the genes cited in this study are causative in GTS, or that they all act alone in GTS etiology, we present convincing weight of evidence that mitochondria are implicated in GTS. Notwithstanding, the deleterious mutation of genes directly involved in mitochondrial dynamics and supply (Figure 4) have the potential to limit neurodevelopment and neurotransmission during periods of peak demand. The exact mitochondrial mechanism implicated in GTS has not been identified as there is no evidence of mitochondrial mediated increases in ROS or ROS related neurodegeneration in GTS as is commonly the case in neurodegenerative disorders. A deficit in neuronal energy supply during development is one possible contributing factor in the etiology of GTS, however, the waning of tic severity in GTS over time appears more consistent with a deficit in neurotransmission possibly compensated for at later ages (185–189). We have no non-molecular evidence of a mitochondrial pathway to GTS at this time, notwithstanding, this is the 1st study to report a mitochondrial pathway to GTS and we are confident it will not be the last. The mitochondrial pathways identified in this study (Figures 2–4) have roles in neuronal circuitry development, synaptic connectivity and neurotransmission (Figure 1) (2, 11). The NTSC and DISC1 and mitochondrial pathways to GTS all intersect around the pivotal role of SAP97 in regulating synaptic signaling downstream of the NTSC and mitochondrial transport downstream of DISC1 thus providing compounding support for a GTS deficit in mitochondrial supply affecting neurotransmission.

## Data Availability Statement

The original contributions generated in the study are included in the article/supplementary materials, further inquiries can be directed to the corresponding author.

## Author Contributions

All authors listed have made a substantial, direct and intellectual contribution to the work, and approved it for publication.

## Conflict of Interest

The authors declare that the research was conducted in the absence of any commercial or financial relationships that could be construed as a potential conflict of interest.

## References

[1] Robertson MM Eapen V Singer HS Martino D Scharf JM Paschou P. Gilles de la Tourette syndrome. Nat Rev Dis Primers. (2017) 3:16097. 10.1038/nrdp.2016.97 28150698

[2] Clarke RA Lee S Eapen V. Pathogenetic model for Tourette syndrome delineates overlap with related neurodevelopmental disorders including Autism. Transl Psychiatry. (2012) 2:e158. 10.1038/tp.2012.75 22948383 PMC3565204

[3] State MW. The genetics of child psychiatric disorders: focus on autism and Tourette syndrome. Neuron. (2010) 68:254–69. 10.1016/j.neuron.2010.10.004 20955933 PMC3292208

[4] O'Rourke JA Scharf JM Yu D Pauls DL. The genetics of Tourette syndrome: a review. J Psychosom Res. (2009) 67:533–45. 10.1016/j.jpsychores.2009.06.006 19913658 PMC2778609

[5] Burd L Li Q Kerbeshian J Klug MG Freeman RD. Tourette syndrome and comorbid pervasive developmental disorders. J Child Neurol. (2009) 24:170–5. 10.1177/0883073808322666 19182154

[6] Green JL Rinehart N Anderson V Nicholson JM Jongeling B Sciberras E. Autism spectrum disorder symptoms in children with ADHD: a community-based study. Res Dev Disabil. (2015) 47:175–84. 10.1016/j.ridd.2015.09.016 26433184

[7] Darrow SM Grados M Sandor P Hirschtritt ME Illmann C Osiecki L. Autism spectrum symptoms in a Tourette's disorder sample. J Am Acad Child Adolesc Psychiatry. (2017) 56:610–7.e1. 10.1016/j.jaac.2017.05.002 28647013 PMC5648014

[8] Kadesjo B Gillberg C. Tourette's disorder: epidemiology and comorbidity in primary school children. J Am Acad Child Adolesc Psychiatry. (2000) 39:548–55. 10.1097/00004583-200005000-00007 10802971

[9] Eapen V Robertson MM. Are there distinct subtypes in Tourette syndrome? Pure-Tourette syndrome versus Tourette syndrome-plus, and simple versus complex tics. Neuropsychiatr Dis Treat. (2015) 11:1431–6. 10.2147/NDT.S72284 26089672 PMC4468986

[10] Clarke RA Eapen V. Balance within the neurexin trans-synaptic connexus stabilizes behavioral control. Front Hum Neurosci. (2014) 8:52. 10.3389/fnhum.2014.00052 24578685 PMC3936185

[11] Fang Z Eapen V Clarke RA. CTNNA3 discordant regulation of nested LRRTM3, implications for autism spectrum disorder and Tourette syndrome. Meta Gene. (2017) 11:43–8. 10.1016/j.mgene.2016.11.003

[12] Verkerk AJ Cath DC van der Linde HC Both J Heutink P Breedveld G. Genetic and clinical analysis of a large Dutch Gilles de la Tourette family. Mol Psychiatry. (2006) 11:954–64. 10.1038/sj.mp.4001877 16894393

[13] Griswold AJ Ma D Cukier HN Nations LD Schmidt MA Chung RH. Evaluation of copy number variations reveals novel candidate genes in autism spectrum disorder-associated pathways. Hum Mol Genet. (2012) 21:3513–23. 10.1093/hmg/dds164 22543975 PMC3392110

[14] Sundberg M Tochitsky I Buchholz DE Winden K Kujala V Kapur K. Purkinje cells derived from TSC patients display hypoexcitability and synaptic deficits associated with reduced FMRP levels and reversed by rapamycin. Mol Psychiatry. (2018) 23:2167–83. 10.1038/s41380-018-0018-4 29449635 PMC6093816

[15] Gazzellone MJ Zhou X Lionel AC Uddin M Thiruvahindrapuram B Liang S. Copy number variation in Han Chinese individuals with autism spectrum disorder. J Neurodev Disord. (2014) 6:34. 10.1186/1866-1955-6-34 25170348 PMC4147384

[16] He WZ Liu WQ Zhong XQ Chen XL Li SY Zhang HM. Analysis of de novo copy number variations in a family affected with autism spectrum disorders using high-resolution array-based comparative genomic hybridization. Zhonghua Yi Xue Yi Chuan Xue Za Zhi. (2012) 29:266–9. 10.3760/cma.j.issn.1003-9406.2012.03.004 22678785

[17] Kalkan Z Durasi IM Sezerman U Atasever-Arslan B. Potential of GRID2 receptor gene for preventing TNF-induced neurodegeneration in autism. Neurosci Lett. (2016) 620:62–9. 10.1016/j.neulet.2016.03.043 27019035

[18] RK CY Merico D Bookman M Jennifer LH Thiruvahindrapuram B Patel RV. Whole genome sequencing resource identifies 18 new candidate genes for autism spectrum disorder. Nat Neurosci. (2017) 20:602–11. 10.1038/nn.4524 28263302 PMC5501701

[19] Turner TN Coe BP Dickel DE Hoekzema K Nelson BJ Zody MC. Genomic patterns of *de novo* mutation in simplex autism. Cell. (2017) 171:710–22.e12. 10.1016/j.cell.2017.08.047 28965761 PMC5679715

[20] Lintas C Picinelli C Piras IS Sacco R Brogna C Persico AM. Copy number variation in 19 Italian multiplex families with autism spectrum disorder: importance of synaptic and neurite elongation genes. Am J Med Genet B Neuropsychiatr Genet. (2017) 174:547–56. 10.1002/ajmg.b.32537 28304131

[21] Turner TN Hormozdiari F Duyzend MH McClymont SA Hook PW Iossifov I. Genome sequencing of autism-affected families reveals disruption of putative noncoding regulatory DNA. Am J Hum Genet. (2016) 98:58–74. 10.1016/j.ajhg.2015.11.023 26749308 PMC4716689

[22] Leblond CS Cliquet F Carton C Huguet G Mathieu A Kergrohen T. Both rare and common genetic variants contribute to autism in the Faroe Islands. NPJ Genom Med. (2019) 4:1. 10.1038/s41525-018-0075-2 30675382 PMC6341098

[23] Schoen M Asoglu H Bauer HF Muller HP Abaei A Sauer AK. Shank3 Transgenic and prenatal zinc-deficient autism mouse models show convergent and individual alterations of brain structures in MRI. Front Neural Circuits. (2019) 13:6. 10.3389/fncir.2019.00006 30853900 PMC6395436

[24] Napoli E Ross-Inta C Song G Wong S Hagerman R Gane LW. Premutation in the fragile X mental retardation 1 (FMR1) gene affects maternal Zn-milk and perinatal brain bioenergetics and scaffolding. Front Neurosci. (2016) 10:159. 10.3389/fnins.2016.00159 27147951 PMC4835505

[25] Clarke RA Fang ZM Diwan AD Gilbert DL. Tourette syndrome and klippel-feil anomaly in a child with chromosome 22q11 duplication. Case Rep Med. (2009) 2009:361518. 10.1155/2009/361518 20069037 PMC2797364

[26] Krishnan V Stoppel DC Nong Y Johnson MA Nadler MJ Ozkaynak E. Autism gene Ube3a and seizures impair sociability by repressing VTA Cbln1. Nature. (2017) 543:507–12. 10.1038/nature21678 28297715 PMC5364052

[27] Lam M Moslem M Bryois J Pronk RJ Uhlin E Ellstrom ID. Single cell analysis of autism patient with bi-allelic NRXN1-alpha deletion reveals skewed fate choice in neural progenitors and impaired neuronal functionality. Exp Cell Res. (2019) 383:111469. 10.1016/j.yexcr.2019.06.014 31302032

[28] Zhang P Lu H Peixoto RT Pines MK Ge Y Oku S. Heparan sulfate organizes neuronal synapses through neurexin partnerships. Cell. (2018) 174:1450–64.e23. 10.1016/j.cell.2018.07.002 30100184 PMC6173057

[29] Kasem E Kurihara T Tabuchi K. Neurexins and neuropsychiatric disorders. Neurosci Res. (2018) 127:53–60. 10.1016/j.neures.2017.10.012 29221905

[30] Gauthier J Siddiqui TJ Huashan P Yokomaku D Hamdan FF Champagne N. Truncating mutations in NRXN2 and NRXN1 in autism spectrum disorders and schizophrenia. Hum Genet. (2011) 130:563–73. 10.1007/s00439-011-0975-z 21424692 PMC3204930

[31] Huang AY Yu D Davis LK Sul JH Tsetsos F Ramensky V. Rare copy number variants in NRXN1 and CNTN6 increase risk for Tourette syndrome. Neuron. (2017) 94:1101–11.e7. 28641109 10.1016/j.neuron.2017.06.010 PMC5568251

[32] Roppongi RT Karimi B Siddiqui TJ. Role of LRRTMs in synapse development and plasticity. Neurosci Res. (2017) 116:18–28. 10.1016/j.neures.2016.10.003 27810425

[33] Harris JJ Jolivet R Attwell D. Synaptic energy use and supply. Neuron. (2012) 75:762–77. 10.1016/j.neuron.2012.08.019 22958818

[34] Raichle ME Gusnard DA. Appraising the brain's energy budget. Proc Natl Acad Sci USA. (2002) 99:10237–9. 10.1073/pnas.172399499 12149485 PMC124895

[35] Bogan N Cabot JB. Light and electron microscopic analyses of intraspinal axon collaterals of sympathetic preganglionic neurons. Brain Res. (1991) 541:241–51. 10.1016/0006-8993(91)91024-U 2054640

[36] Mutsaers SE Carroll WM. Focal accumulation of intra-axonal mitochondria in demyelination of the cat optic nerve. Acta Neuropathol. (1998) 96:139–43. 10.1007/s004010050873 9705128

[37] Fabricius C Berthold CH Rydmark M. Axoplasmic organelles at nodes of Ranvier. II. Occurrence and distribution in large myelinated spinal cord axons of the adult cat. J Neurocytol. (1993) 22l:941–54. 10.1007/BF01218352 7507976

[38] Shepherd GM Harris KM. Three-dimensional structure and composition of CA3–>CA1 axons in rat hippocampal slices: implications for presynaptic connectivity and compartmentalization. J Neurosci. (1998) 18:8300–10. 10.1523/JNEUROSCI.18-20-08300.1998 9763474 PMC6792846

[39] Flippo KH Strack S. An emerging role for mitochondrial dynamics in schizophrenia. Schizophr Res. (2017) 187:26–32. 10.1016/j.schres.2017.05.003 28526279 PMC5646380

[40] Kimura T Murakami F. Evidence that dendritic mitochondria negatively regulate dendritic branching in pyramidal neurons in the neocortex. J Neurosci. (2014) 34:6938–51. 10.1523/JNEUROSCI.5095-13.2014 24828647 PMC6608104

[41] Chaturvedi RK Flint Beal M. Mitochondrial diseases of the brain. Free Radic Biol Med. (2013) 63:1–29. 10.1016/j.freeradbiomed.2013.03.018 23567191

[42] Adzic M Brkic Z Bulajic S Mitic M Radojcic MB. Antidepressant action on mitochondrial dysfunction in psychiatric disorders. Drug Dev Res. (2016) 77:400–6. 10.1002/ddr.21332 27539538

[43] Bergman O Ben-Shachar D. Mitochondrial oxidative phosphorylation system (OXPHOS) deficits in schizophrenia: possible interactions with cellular processes. Can J Psychiatry. (2016) 61:457–69. 10.1177/0706743716648290 27412728 PMC4959648

[44] Machado AK Pan AY da Silva TM Duong A Andreazza AC. Upstream pathways controlling mitochondrial function in major psychosis: a focus on bipolar disorder. Can J Psychiatry. (2016) 61:446–56. 10.1177/0706743716648297 27310240 PMC4959649

[45] Curtis D Brett P Dearlove AM McQuillin A Kalsi G Robertson MM. Genome scan of Tourette syndrome in a single large pedigree shows some support for linkage to regions of chromosomes 5, 10 and 13. Psychiatr Genet. (2004) 14:83–7. 10.1097/01.ypg.0000107927.32051.f5 15167693

[46] Zhang H Leckman JF Pauls DL Tsai CP Kidd KK Campos MR. Genomewide scan of hoarding in sib pairs in which both sibs have Gilles de la Tourette syndrome. Am J Hum Genet. (2002) 70:896–904. 10.1086/339520 11840360 PMC379118

[47] Simonic I Gericke GS Ott J Weber JL. Identification of genetic markers associated with Gilles de la Tourette syndrome in an Afrikaner population. Am J Hum Genet. (1998) 63:839–46. 10.1086/302002 9718333 PMC1377391

[48] A full genome screen for autism with evidence for linkage to a region on chromosome 7q. International molecular genetic study of autism consortium. Hum Mol Genet. (1998) 7:571–8. 10.1093/hmg/7.3.571 9546821

[49] Barrett S Beck JC Bernier R Bisson E Braun TA Casavant TL. An autosomal genomic screen for autism. Collaborative linkage study of autism. Am J Med Genet. (1999) 88:609–15. 10.1002/(SICI)1096-8628(19991215)88:6<609::AID-AJMG7>3.0.CO;2-L 10581478

[50] Shao Y Wolpert CM Raiford KL Menold MM Donnelly SL Ravan SA. Genomic screen and follow-up analysis for autistic disorder. Am J Med Genet. (2002) 114:99–105. 10.1002/ajmg.10153 11840513

[51] Schellenberg GD Dawson G Sung YJ Estes A Munson J Rosenthal E. Evidence for multiple loci from a genome scan of autism kindreds. Mol Psychiatry. (2006) 11:1049–60. 10.1038/sj.mp.4001874 16880825

[52] Maestrini E Pagnamenta AT Lamb JA Bacchelli E Sykes NH Sousa I. High-density SNP association study and copy number variation analysis of the AUTS1 and AUTS5 loci implicate the IMMP2L-DOCK4 gene region in autism susceptibility. Mol Psychiatry. (2010) 15:954–68. 10.1038/mp.2009.34 19401682 PMC2934739

[53] Tourette Syndrome Association International Consortium for G. Genome scan for Tourette disorder in affected-sibling-pair and multigenerational families. Am J Hum Genet. (2007) 80:265–72. 10.1086/511052 17304708 PMC1785345

[54] Suarez-Rama JJ Arrojo M Sobrino B Amigo J Brenlla J Agra S. Resequencing and association analysis of coding regions at twenty candidate genes suggest a role for rare risk variation at AKAP9 and protective variation at NRXN1 in schizophrenia susceptibility. J Psychiatr Res. (2015) 66, 67:38–44. 10.1016/j.jpsychires.2015.04.013 25943950

[55] Lintas C Sacco R Garbett K Mirnics K Militerni R Bravaccio C. Involvement of the PRKCB1 gene in autistic disorder: significant genetic association and reduced neocortical gene expression. Mol Psychiatry. (2009) 14:705–18. 10.1038/mp.2008.21 18317465

[56] Philippi A Roschmann E Tores F Lindenbaum P Benajou A Germain-Leclerc L. Haplotypes in the gene encoding protein kinase c-beta (PRKCB1) on chromosome 16 are associated with autism. Mol Psychiatry. (2005) 10:950–60. 10.1038/sj.mp.4001704 16027742

[57] Eicher JD Gruen JR. Language impairment and dyslexia genes influence language skills in children with autism spectrum disorders. Autism Res. (2015) 8:229–34. 10.1002/aur.1436 25448322 PMC4412753

[58] Wang S Mandell JD Kumar Y Sun N Morris MT Arbelaez J. *De novo* sequence and copy number variants are strongly associated with tourette disorder and implicate cell polarity in pathogenesis. Cell Rep. (2018) 25:3544. 10.1016/j.celrep.2018.12.024 30566877

[59] Malhotra D Sebat J. CNVs: harbingers of a rare variant revolution in psychiatric genetics. Cell. (2012) 148:1223–41. 10.1016/j.cell.2012.02.039 22424231 PMC3351385

[60] Johnstone M Maclean A Heyrman L Lenaerts AS Nordin A Nilsson LG. Copy number variations in DISC1 and DISC1-interacting partners in major mental illness. Mol Neuropsychiatry. (2015) 1:175–90. 10.1159/000438788 27239468 PMC4872463

[61] Fernandez TV Sanders SJ Yurkiewicz IR Ercan-Sencicek AG Kim YS Fishman DO. Rare copy number variants in tourette syndrome disrupt genes in histaminergic pathways and overlap with autism. Biol Psychiatry. (2012) 71:392–402. 10.1016/j.biopsych.2011.09.034 22169095 PMC3282144

[62] McGrath LM Yu D Marshall C Davis LK Thiruvahindrapuram B Li B. Copy number variation in obsessive-compulsive disorder and tourette syndrome: a cross-disorder study. J Am Acad Child Adolesc Psychiatry. (2014) 53:910–9. 10.1016/j.jaac.2014.04.022 25062598 PMC4218748

[63] Sundaram SK Huq AM Wilson BJ Chugani HT. Tourette syndrome is associated with recurrent exonic copy number variants. Neurology. (2010) 74:1583–90. 10.1212/WNL.0b013e3181e0f147 20427753 PMC2876824

[64] Bertelsen B Melchior L Jensen LR Groth C Glenthoj B Rizzo R. Intragenic deletions affecting two alternative transcripts of the IMMP2L gene in patients with Tourette syndrome. Eur J Hum Genet. (2014) 22:1283–9. 10.1038/ejhg.2014.24 24549057 PMC4200436

[65] Elia J Gai X Xie HM Perin JC Geiger E Glessner JT. Rare structural variants found in attention-deficit hyperactivity disorder are preferentially associated with neurodevelopmental genes. Mol Psychiatry. (2010) 15:637–46. 10.1038/mp.2009.57 19546859 PMC2877197

[66] Jang W Kim Y Han E Park J Chae H Kwon A. Chromosomal microarray analysis as a first-tier clinical diagnostic test in patients with developmental delay/intellectual disability, autism spectrum disorders, and multiple congenital anomalies: a prospective multicenter study in Korea. Ann Lab Med. (2019) 39:299–310. 10.3343/alm.2019.39.3.299 30623622 PMC6340852

[67] Sundaram SK Huq AM Sun Z Yu W Bennett L Wilson BJ. Exome sequencing of a pedigree with Tourette syndrome or chronic tic disorder. Ann Neurol. (2011) 69:901–4. 10.1002/ana.22398 21520241

[68] Wittkowski KM Sonakya V Bigio B Tonn MK Shic F Ascano M. A novel computational biostatistics approach implies impaired dephosphorylation of growth factor receptors as associated with severity of autism. Transl Psychiatry. (2014) 4:e354. 10.1038/tp.2013.124 24473445 PMC3905234

[69] Wang L Jia P Wolfinger RD Chen X Zhao Z. Gene set analysis of genome-wide association studies: methodological issues and perspectives. Genomics. (2011) 98:1–8. 10.1016/j.ygeno.2011.04.006 21565265 PMC3852939

[70] de Leeuw C Goudriaan A Smit AB Yu D Mathews CA Scharf JM. Involvement of astrocyte metabolic coupling in Tourette syndrome pathogenesis. Eur J Hum Genet. (2015) 23:1519–22. 10.1038/ejhg.2015.22 25735483 PMC4613465

[71] Casey JP Magalhaes T Conroy JM Regan R Shah N Anney R. A novel approach of homozygous haplotype sharing identifies candidate genes in autism spectrum disorder. Hum Genet. (2012) 131:565–79. 10.1007/s00439-011-1094-6 21996756 PMC3303079

[72] Boghosian-Sell L Comings DE Overhauser J. Tourette syndrome in a pedigree with a 7;18 translocation: identification of a YAC spanning the translocation breakpoint at 18q22.3. Am J Hum Genet. (1996) 59:999–1005. 8900226 PMC1914824

[73] Petek E Windpassinger C Vincent JB Cheung J Boright AP Scherer SW. Disruption of a novel gene (IMMP2L) by a breakpoint in 7q31 associated with Tourette syndrome. Am J Hum Genet. (2001) 68:848–58. 10.1086/319523 11254443 PMC1275638

[74] Zhang Y Liu Y Zarrei M Tong W Dong R Wang Y. Association of IMMP2L deletions with autism spectrum disorder: a trio family study and meta-analysis. Am J Med Genet B Neuropsychiatr Genet. (2018) 177:93–100. 10.1002/ajmg.b.32608 29152845

[75] Patel C Cooper-Charles L McMullan DJ Walker JM Davison V Morton J. Translocation breakpoint at 7q31 associated with tics: further evidence for IMMP2L as a candidate gene for Tourette syndrome. Eur J Hum Genet. (2011) 19:634–9. 10.1038/ejhg.2010.238 21386874 PMC3110039

[76] Robertson MM Shelley BP Dalwai S Brewer C Critchley HD. A patient with both Gilles de la Tourette's syndrome and chromosome 22q11 deletion syndrome: clue to the genetics of Gilles de la Tourette's syndrome? J Psychosom Res. (2006) 61:365–8. 10.1016/j.jpsychores.2006.06.011 16938515

[77] Tang G Gutierrez Rios P Kuo SH Akman HO Rosoklija G Tanji K. Mitochondrial abnormalities in temporal lobe of autistic brain. Neurobiol Dis. (2013) 54:349–61. 10.1016/j.nbd.2013.01.006 23333625 PMC3959772

[78] Anitha A Nakamura K Thanseem I Matsuzaki H Miyachi T Tsujii M. Downregulation of the expression of mitochondrial electron transport complex genes in autism brains. Brain Pathol. (2013) 23:294–302. 10.1111/bpa.12002 23088660 PMC8029398

[79] Anitha A Nakamura K Thanseem I Yamada K Iwayama Y Toyota T. Brain region-specific altered expression and association of mitochondria-related genes in autism. Mol Autism. (2012) 3:12. 10.1186/2040-2392-3-12 23116158 PMC3528421

[80] Voineagu I. Gene expression studies in autism: moving from the genome to the transcriptome and beyond. Neurobiol Dis. (2012) 45:69–75. 10.1016/j.nbd.2011.07.017 21839838

[81] Schwede M Nagpal S Gandal MJ Parikshak NN Mirnics K Geschwind DH. Strong correlation of downregulated genes related to synaptic transmission and mitochondria in post-mortem autism cerebral cortex. J Neurodev Disord. (2018) 10:18. 10.1186/s11689-018-9237-x 29859039 PMC5984825

[82] Ji L Chauhan A Chauhan V. Reduced activity of protein kinase C in the frontal cortex of subjects with regressive autism: relationship with developmental abnormalities. Int J Biol Sci. (2012) 8:1075–84. 10.7150/ijbs.4742 22949890 PMC3432855

[83] Shen M Wang F Li M Sah N Stockton ME Tidei JJ. Reduced mitochondrial fusion and Huntingtin levels contribute to impaired dendritic maturation and behavioral deficits in Fmr1-mutant mice. Nat Neurosci. (2019) 22:386–400. 10.1038/s41593-019-0338-y 30742117 PMC6556892

[84] Lu B Poirier C Gaspar T Gratzke C Harrison W Busija D. A mutation in the inner mitochondrial membrane peptidase 2-like gene (Immp2l) affects mitochondrial function and impairs fertility in mice. Biol Reprod. (2008) 78:601–10. 10.1095/biolreprod.107.065987 18094351

[85] Kreilaus F Chesworth R Eapen V Clarke R Karl T. First behavioural assessment of a novel Immp2l knockdown mouse model with relevance for Gilles de la Tourette syndrome and Autism spectrum disorder. Behav Brain Res. (2019) 374:112057. 10.1016/j.bbr.2019.112057 31233820

[86] Norkett R Modi S Kittler JT. Mitochondrial roles of the psychiatric disease risk factor DISC1. Schizophr Res. (2017) 187:47–54. 10.1016/j.schres.2016.12.025 28087269

[87] Hirokawa N Niwa S Tanaka Y. Molecular motors in neurons: transport mechanisms and roles in brain function, development, and disease. Neuron. (2010) 68:610–38. 10.1016/j.neuron.2010.09.039 21092854

[88] Kanai Y Okada Y Tanaka Y Harada A Terada S Hirokawa N. KIF5C, a novel neuronal kinesin enriched in motor neurons. J Neurosci. (2000) 20:6374–84. 10.1523/JNEUROSCI.20-17-06374.2000 10964943 PMC6772948

[89] Iworima DG Pasqualotto BA Rintoul GL. Kif5 regulates mitochondrial movement, morphology, function and neuronal survival. Mol Cell Neurosci. (2016) 72:22–33. 10.1016/j.mcn.2015.12.014 26767417

[90] Duis J Dean S Applegate C Harper A Xiao R He W. KIF5A mutations cause an infantile onset phenotype including severe myoclonus with evidence of mitochondrial dysfunction. Ann Neurol. (2016) 80:633–7. 10.1002/ana.24744 27463701 PMC5042851

[91] Campbell PD Shen K Sapio MR Glenn TD Talbot WS Marlow FL. Unique function of Kinesin Kif5A in localization of mitochondria in axons. J Neurosci. (2014) 34:14717–32. 10.1523/JNEUROSCI.2770-14.2014 25355224 PMC4212069

[92] Guo Q Liao S Zhu Z Li Y Li F Xu C. Structural basis for the recognition of kinesin family member 21A (KIF21A) by the ankyrin domains of KANK1 and KANK2 proteins. J Biol Chem. (2018) 293:557–66. 10.1074/jbc.M117.817494 29183992 PMC5767861

[93] King MD Phillips GW Bignone PA Hayes NV Pinder JC Baines AJ. A conserved sequence in calmodulin regulated spectrin-associated protein 1 links its interaction with spectrin and calmodulin to neurite outgrowth. J Neurochem. (2014) 128:391–402. .org/10.1111/jnc.12462 24117850 10.1111/jnc.12462 PMC4016758

[94] Vos M Lauwers E Verstreken P. Synaptic mitochondria in synaptic transmission and organization of vesicle pools in health and disease. Front Synaptic Neurosci. (2010) 2:139. 10.3389/fnsyn.2010.00139 21423525 PMC3059669

[95] Sun T Qiao H Pan PY Chen Y Sheng ZH. Motile axonal mitochondria contribute to the variability of presynaptic strength. Cell Rep. (2013) 4:413–9. 10.1016/j.celrep.2013.06.040 23891000 PMC3757511

[96] Picard M McEwen BS. Mitochondria impact brain function and cognition. Proc Natl Acad Sci USA. (2014) 111:7–8. 10.1073/pnas.1321881111 24367081 PMC3890847

[97] Hara Y Yuk F Puri R Janssen WG Rapp PR Morrison JH. Presynaptic mitochondrial morphology in monkey prefrontal cortex correlates with working memory and is improved with estrogen treatment. Proc Natl Acad Sci USA. (2014) 111:486–91. 10.1073/pnas.1311310110 24297907 PMC3890848

[98] Schwarz TL. Mitochondrial trafficking in neurons. Cold Spring Harb Perspect Biol. (2013) 5. 10.1101/cshperspect.a011304 PMC3660831 23732472

[99] Kang M Akbarali HI. Denitration of L-type calcium channel. FEBS Lett. (2008) 582:3033–6. 10.1016/j.febslet.2008.07.042 18675806 PMC2533528

[100] Cai Q Sheng ZH. Moving or stopping mitochondria: miro as a traffic cop by sensing calcium. Neuron. (2009) 61:493–6. 10.1016/j.neuron.2009.02.003 19249268 PMC5102254

[101] Saxton WM Hollenbeck PJ. The axonal transport of mitochondria. J Cell Sci. (2012) 125:2095–104. 10.1242/jcs.053850 22619228 PMC3656622

[102] Ashrafi G Schwarz TL. The pathways of mitophagy for quality control and clearance of mitochondria. Cell Death Differ. (2013) 20:31–42. 10.1038/cdd.2012.81 22743996 PMC3524633

[103] MacAskill AF Brickley K Stephenson FA Kittler JT. GTPase dependent recruitment of Grif-1 by Miro1 regulates mitochondrial trafficking in hippocampal neurons. Mol Cell Neurosci. (2009) 40:301–12. 10.1016/j.mcn.2008.10.016 19103291

[104] van Spronsen M Mikhaylova M Lipka J Schlager MA van den Heuvel DJ Kuijpers M. TRAK/Milton motor-adaptor proteins steer mitochondrial trafficking to axons and dendrites. Neuron. (2013) 77:485–502. 10.1016/j.neuron.2012.11.027 23395375

[105] Loss O Stephenson FA. Localization of the kinesin adaptor proteins trafficking kinesin proteins 1 and 2 in primary cultures of hippocampal pyramidal and cortical neurons. J Neurosci Res. (2015) 93:1056–66. 10.1002/jnr.23549 25653102

[106] Brickley K Stephenson FA. Trafficking kinesin protein (TRAK)-mediated transport of mitochondria in axons of hippocampal neurons. J Biol Chem. (2011) 286:18079–92. 10.1074/jbc.M111.236018 21454691 PMC3093881

[107] Nguyen TT Oh SS Weaver D Lewandowska A Maxfield D Schuler MH. Loss of Miro1-directed mitochondrial movement results in a novel murine model for neuron disease. Proc Natl Acad Sci USA. (2014) 111:E3631–40. 10.1073/pnas.1402449111 25136135 PMC4156725

[108] Millar JK James R Christie S Porteous DJ. Disrupted in schizophrenia 1 (DISC1): subcellular targeting and induction of ring mitochondria. Mol Cell Neurosci. (2005) 30:477–84. 10.1016/j.mcn.2005.08.021 16209927

[109] Park YU Jeong J Lee H Mun JY Kim JH Lee JS. Disrupted-in-schizophrenia 1 (DISC1) plays essential roles in mitochondria in collaboration with Mitofilin. Proc Natl Acad Sci USA. (2010) 107:17785–90. 10.1073/pnas.1004361107 20880836 PMC2955093

[110] Tsuboi D Kuroda K Tanaka M Namba T Iizuka Y Taya S. Disrupted-in-schizophrenia 1 regulates transport of ITPR1 mRNA for synaptic plasticity. Nat Neurosci. (2015) 18:698–707. 10.1038/nn.3984 25821909

[111] Kijima K Numakura C Izumino H Umetsu K Nezu A Shiiki T. Mitochondrial GTPase mitofusin 2 mutation in Charcot-Marie-Tooth neuropathy type 2A. Hum Genet. (2005) 116:23–7. 10.1007/s00439-004-1199-2 15549395

[112] Ogawa F Murphy LC Malavasi EL O'Sullivan ST Torrance HS Porteous DJ. NDE1 and GSK3beta associate with TRAK1 and regulate axonal mitochondrial motility: identification of cyclic AMP as a novel modulator of axonal mitochondrial trafficking. ACS Chem Neurosci. (2016) 7:553–64. 10.1021/acschemneuro.5b00255 26815013

[113] Atkin TA MacAskill AF Brandon NJ Kittler JT. Disrupted in Schizophrenia-1 regulates intracellular trafficking of mitochondria in neurons. Mol Psychiatry. (2011) 16:122–4. 10.1038/mp.2010.110 21079610

[114] Norkett R Modi S Birsa N Atkin TA Ivankovic D Pathania M. DISC1-dependent regulation of mitochondrial dynamics controls the morphogenesis of complex neuronal dendrites. J Biol Chem. (2016) 291:613–29. 10.1074/jbc.M115.699447 26553875 PMC4705382

[115] Amiott EA Lott P Soto J Kang PB McCaffery JM DiMauro S. Mitochondrial fusion and function in Charcot-Marie-Tooth type 2A patient fibroblasts with mitofusin 2 mutations. Exp Neurol. (2008) 211:115–27. 10.1016/j.expneurol.2008.01.010 18316077 PMC2409111

[116] Feng Y Walsh CA. Protein-protein interactions, cytoskeletal regulation and neuronal migration. Nat Rev Neurosci. (2001) 2:408–16. 10.1038/35077559 11389474

[117] Feng Y Walsh CA. Mitotic spindle regulation by Nde1 controls cerebral cortical size. Neuron. (2004) 44:279–93. 10.1016/j.neuron.2004.09.023 15473967

[118] Alkuraya FS Cai X Emery C Mochida GH Al-Dosari MS Felie JM. Human mutations in NDE1 cause extreme microcephaly with lissencephaly [corrected]. Am J Hum Genet. (2011) 88:536–47. 10.1016/j.ajhg.2011.04.003 21529751 PMC3146728

[119] Bradshaw NJ Ogawa F Antolin-Fontes B Chubb JE Carlyle BC Christie S. DISC1, PDE4B, and NDE1 at the centrosome and synapse. Biochem Biophys Res Commun. (2008) 377:1091–6. 10.1016/j.bbrc.2008.10.120 18983980

[120] Dasgupta B Milbrandt J. AMP-activated protein kinase phosphorylates retinoblastoma protein to control mammalian brain development. Dev Cell. (2009) 16:256–70. 10.1016/j.devcel.2009.01.005 19217427 PMC2662481

[121] Williams T Courchet J Viollet B Brenman JE Polleux F. AMP-activated protein kinase (AMPK) activity is not required for neuronal development but regulates axogenesis during metabolic stress. Proc Natl Acad Sci USA. (2011) 108:5849–54. 10.1073/pnas.1013660108 21436046 PMC3078367

[122] Ji L Chauhan V Flory MJ Chauhan A. Brain region-specific decrease in the activity and expression of protein kinase A in the frontal cortex of regressive autism. PLoS ONE. (2011) 6:e23751. 10.1371/journal.pone.0023751 21909354 PMC3166116

[123] Cianfrocco MA DeSantis ME Leschziner AE Reck-Peterson SL. Mechanism and regulation of cytoplasmic dynein. Annu Rev Cell Dev Biol. (2015) 31:83–108. 10.1146/annurev-cellbio-100814-125438 26436706 PMC4644480

[124] Reddy BJ Mattson M Wynne CL Vadpey O Durra A Chapman D. Load-induced enhancement of Dynein force production by LIS1-NudE *in vivo* and *in vitro*. Nat Commun. (2016) 7:12259. 10.1038/ncomms12259 27489054 PMC4976208

[125] Bradshaw NJ Soares DC Carlyle BC Ogawa F Davidson-Smith H Christie S. PKA phosphorylation of NDE1 is DISC1/PDE4 dependent and modulates its interaction with LIS1 and NDEL1. J Neurosci. (2011) 31:9043–54. 10.1523/JNEUROSCI.5410-10.2011 21677187 PMC3610090

[126] Slater PG Cammarata GM Samuelson AG Magee A Hu Y Lowery LA. XMAP215 promotes microtubule-F-actin interactions to regulate growth cone microtubules during axon guidance. J Cell Sci. (2019) 132:1–14. 10.1242/jcs.224311 30890650 PMC6526707

[127] Sears JC Choi WJ Broadie K. Fragile X Mental Retardation Protein positively regulates PKA anchor Rugose and PKA activity to control actin assembly in learning/memory circuitry. Neurobiol Dis. (2019) 127:53–64. 10.1016/j.nbd.2019.02.004 30771457 PMC6588493

[128] Tao K Matsuki N Koyama R. AMP-activated protein kinase mediates activity-dependent axon branching by recruiting mitochondria to axon. Dev Neurobiol. (2014) 74:557–73. 10.1002/dneu.22149 24218086

[129] Beurel E Grieco SF Jope RS. Glycogen synthase kinase-3 (GSK3): regulation, actions, and diseases. Pharmacol Ther. (2015) 148:114–31. 10.1016/j.pharmthera.2014.11.016 25435019 PMC4340754

[130] Bijur GN Jope RS. Glycogen synthase kinase-3 beta is highly activated in nuclei and mitochondria. Neuroreport. (2003) 14:2415–9. 10.1097/00001756-200312190-00025 14663202

[131] Nishihara M Miura T Miki T Tanno M Yano T Naitoh K. Modulation of the mitochondrial permeability transition pore complex in GSK-3beta-mediated myocardial protection. J Mol Cell Cardiol. (2007) 43:564–70. 10.1016/j.yjmcc.2007.08.010 17931653

[132] Boccitto M Doshi S Newton IP Nathke I Neve R Dong F. Opposing actions of the synapse-associated protein of 97-kDa molecular weight (SAP97) and Disrupted in Schizophrenia 1 (DISC1) on Wnt/beta-catenin signaling. Neuroscience. (2016) 326:22–30. 10.1016/j.neuroscience.2016.03.048 27026592 PMC4853273

[133] Bijur GN Jope RS. Rapid accumulation of Akt in mitochondria following phosphatidylinositol 3-kinase activation. J Neurochem. (2003) 87:1427–35. 10.1046/j.1471-4159.2003.02113.x 14713298 PMC2040497

[134] King TD Clodfelder-Miller B Barksdale KA Bijur GN. Unregulated mitochondrial GSK3beta activity results in NADH: ubiquinone oxidoreductase deficiency. Neurotox Res. (2008) 14:367–82. 10.1007/BF03033861 19073440 PMC2677990

[135] Chen H Chan DC. Mitochondrial dynamics–fusion, fission, movement, and mitophagy–in neurodegenerative diseases. Hum Mol Genet. (2009) 18:R169–76. 10.1093/hmg/ddp326 19808793 PMC2758711

[136] Ishihara N Otera H Oka T Mihara K. Regulation and physiologic functions of GTPases in mitochondrial fusion and fission in mammals. Antioxid Redox Signal. (2013) 19:389–99. 10.1089/ars.2012.4830 22871170

[137] Schrepfer E Scorrano L. Mitofusins, from Mitochondria to Metabolism. Mol Cell. (2016) 61:683–94. 10.1016/j.molcel.2016.02.022 26942673

[138] Cartoni R Martinou JC. Role of mitofusin 2 mutations in the physiopathology of Charcot-Marie-Tooth disease type 2A. Exp Neurol. (2009) 218:268–73. 10.1016/j.expneurol.2009.05.003 19427854

[139] Zuchner S Mersiyanova IV Muglia M Bissar-Tadmouri N Rochelle J Dadali EL. Mutations in the mitochondrial GTPase mitofusin 2 cause Charcot-Marie-Tooth neuropathy type 2A. Nat Genet. (2004) 36:449–51. 10.1038/ng1341 15064763

[140] Baloh RH Schmidt RE Pestronk A Milbrandt J. Altered axonal mitochondrial transport in the pathogenesis of Charcot-Marie-Tooth disease from mitofusin 2 mutations. J Neurosci. (2007) 27:422–30. 10.1523/JNEUROSCI.4798-06.2007 17215403 PMC6672077

[141] Misko A Jiang S Wegorzewska I Milbrandt J Baloh RH. Mitofusin 2 is necessary for transport of axonal mitochondria and interacts with the Miro/Milton complex. J Neurosci. (2010) 30:4232–40. 10.1523/JNEUROSCI.6248-09.2010 20335458 PMC2852190

[142] Chen H McCaffery JM Chan DC. Mitochondrial fusion protects against neurodegeneration in the cerebellum. Cell. (2007) 130:548–62. 10.1016/j.cell.2007.06.026 17693261

[143] Chen H Detmer SA Ewald AJ Griffin EE Fraser SE Chan DC. Mitofusins Mfn1 and Mfn2 coordinately regulate mitochondrial fusion and are essential for embryonic development. J Cell Biol. (2003) 160:189–200. 10.1083/jcb.200211046 12527753 PMC2172648

[144] Chapman AL Bennett EJ Ramesh TM De Vos KJ Grierson AJ. Axonal transport defects in a mitofusin 2 loss of function model of charcot-marie-tooth disease in Zebrafish. PLoS ONE. (2013) 8:e67276. 10.1371/journal.pone.0067276 23840650 PMC3694133

[145] Lenaers G Hamel C Delettre C Amati-Bonneau P Procaccio V Bonneau D. Dominant optic atrophy. Orphanet J Rare Dis. (2012) 7:46. 10.1186/1750-1172-7-46 22776096 PMC3526509

[146] Yu-Wai-Man P Griffiths PG Gorman GS Lourenco CM Wright AF Auer-Grumbach M. Multi-system neurological disease is common in patients with OPA1 mutations. Brain. (2010) 133:771–86. 10.1093/brain/awq007 20157015 PMC2842512

[147] Chao de la Barca JM Prunier-Mirebeau D Amati-Bonneau P Ferre M Sarzi E Bris C. OPA1-related disorders: diversity of clinical expression, modes of inheritance and pathophysiology. Neurobiol Dis. (2016) 90:20–6. 10.1016/j.nbd.2015.08.015 26311407

[148] Davies VJ Hollins AJ Piechota MJ Yip W Davies JR White KE. Opa1 deficiency in a mouse model of autosomal dominant optic atrophy impairs mitochondrial morphology, optic nerve structure and visual function. Hum Mol Genet. (2007) 16:1307–18. 10.1093/hmg/ddm079 17428816

[149] Bertholet AM Millet AM Guillermin O Daloyau M Davezac N Miquel MC. OPA1 loss of function affects *in vitro* neuronal maturation. Brain. (2013) 136:1518–33. 10.1093/brain/awt060 23543485

[150] Otera H Ishihara N Mihara K. New insights into the function and regulation of mitochondrial fission. Biochim Biophys Acta. (2013) 1833:1256–68. 10.1016/j.bbamcr.2013.02.002 23434681

[151] Sheffer R Douiev L Edvardson S Shaag A Tamimi K Soiferman D. Postnatal microcephaly and pain insensitivity due to a *de novo* heterozygous DNM1L mutation causing impaired mitochondrial fission and function. Am J Med Genet A. (2016) 170:1603–7. 10.1002/ajmg.a.37624 26992161

[152] Waterham HR Koster J van Roermund CW Mooyer PA Wanders RJ Leonard JV. A lethal defect of mitochondrial and peroxisomal fission. N Engl J Med. (2007) 356:1736–41. 10.1056/NEJMoa064436 17460227

[153] Fahrner JA Liu R Perry MS Klein J Chan DC. A novel *de novo* dominant negative mutation in DNM1L impairs mitochondrial fission and presents as childhood epileptic encephalopathy. Am J Med Genet A. (2016) 170:2002–11. 10.1002/ajmg.a.37721 27145208 PMC5100740

[154] Nasca A Legati A Baruffini E Nolli C Moroni I Ardissone A. Biallelic mutations in DNM1L are associated with a slowly progressive infantile encephalopathy. Hum Mutat. (2016) 37:898–903. 10.1002/humu.23033 27328748 PMC5108486

[155] Vanstone JR Smith AM McBride S Naas T Holcik M Antoun G. DNM1L-related mitochondrial fission defect presenting as refractory epilepsy. Eur J Hum Genet. (2016) 24:1084–8. 10.1038/ejhg.2015.243 26604000 PMC5070894

[156] Yoon G Malam Z Paton T Marshall CR Hyatt E Ivakine Z. Lethal disorder of mitochondrial fission caused by mutations in DNM1L. J Pediatr. (2016) 171:313–6.e1-2. 10.1016/j.jpeds.2015.12.060 26825290

[157] Oettinghaus B Schulz JM Restelli LM Licci M Savoia C Schmidt A. Synaptic dysfunction, memory deficits and hippocampal atrophy due to ablation of mitochondrial fission in adult forebrain neurons. Cell Death Differ. (2016) 23:18–28. 10.1038/cdd.2015.39 25909888 PMC4815974

[158] Shields LY Kim H Zhu L Haddad D Berthet A Pathak D. Dynamin-related protein 1 is required for normal mitochondrial bioenergetic and synaptic function in CA1 hippocampal neurons. Cell Death Dis. (2015) 6:e1725. 10.1038/cddis.2015.94 25880092 PMC4650558

[159] Ishihara N Nomura M Jofuku A Kato H Suzuki SO Masuda K. Mitochondrial fission factor Drp1 is essential for embryonic development and synapse formation in mice. Nat Cell Biol. (2009) 11:958–66. 10.1038/ncb1907 19578372

[160] Wakabayashi J Zhang Z Wakabayashi N Tamura Y Fukaya M Kensler TW. The dynamin-related GTPase Drp1 is required for embryonic and brain development in mice. J Cell Biol. (2009) 186:805–16. 10.1083/jcb.200903065 19752021 PMC2753156

[161] Koch A Thiemann M Grabenbauer M Yoon Y McNiven MA Schrader M. Dynamin-like protein 1 is involved in peroxisomal fission. J Biol Chem. (2003) 278:8597–605. 10.1074/jbc.M211761200 12499366

[162] Chang CR Blackstone C. Cyclic AMP-dependent protein kinase phosphorylation of Drp1 regulates its GTPase activity and mitochondrial morphology. J Biol Chem. (2007) 282:21583–7. 10.1074/jbc.C700083200 17553808

[163] Cribbs JT Strack S. Reversible phosphorylation of Drp1 by cyclic AMP-dependent protein kinase and calcineurin regulates mitochondrial fission and cell death. EMBO Rep. (2007) 8:939–44. 10.1038/sj.embor.7401062 17721437 PMC2002551

[164] Wong H Levenga J Cain P Rothermel B Klann E Hoeffer C. RCAN1 overexpression promotes age-dependent mitochondrial dysregulation related to neurodegeneration in Alzheimer's disease. Acta Neuropathol. (2015) 130:829–43. 10.1007/s00401-015-1499-8 26497675 PMC4782929

[165] Parra V Altamirano F Hernandez-Fuentes CP Tong D Kyrychenko V Rotter D. Down syndrome critical region 1 gene, Rcan1, helps maintain a more fused mitochondrial network. Circ Res. (2018) 122:e20–33. 10.1161/CIRCRESAHA.117.311522 29362227 PMC5924463

[166] Mizuguchi T Nakashima M Kato M Okamoto N Kurahashi H Ekhilevitch N. Loss-of-function and gain-of-function mutations in PPP3CA cause two distinct disorders. Hum Mol Genet. (2018) 27:1421–33. 10.1093/hmg/ddy052 29432562

[167] Toyama EQ Herzig S Courchet J Lewis TL Jr. Loson OC Hellberg K. Metabolism. AMP-activated protein kinase mediates mitochondrial fission in response to energy stress. Science. (2016) 351:275–81. 10.1126/science.aab4138 26816379 PMC4852862

[168] Yu R Liu T Jin SB Ning C Lendahl U Nister M. MIEF1/2 function as adaptors to recruit Drp1 to mitochondria and regulate the association of Drp1 with Mff. Sci Rep. (2017) 7:880. 10.1038/s41598-017-00853-x 28408736 PMC5429825

[169] Lee JE Westrate LM Wu H Page C Voeltz GK. Multiple dynamin family members collaborate to drive mitochondrial division. Nature. (2016) 540:139–43. 10.1038/nature20555 27798601 PMC5656044

[170] Piggott LA Bauman AL Scott JD Dessauer CW. The A-kinase anchoring protein Yotiao binds and regulates adenylyl cyclase in brain. Proc Natl Acad Sci USA. (2008) 105:13835–40. 10.1073/pnas.0712100105 18772391 PMC2544540

[171] Sanderson JL Scott JD Dell'Acqua ML. Control of homeostatic synaptic plasticity by AKAP-anchored kinase and phosphatase regulation of Ca(2+)-Permeable AMPA receptors. J Neurosci. (2018) 38:2863–76. 10.1523/JNEUROSCI.2362-17.2018 29440558 PMC5852664

[172] Murphy JG Sanderson JL Gorski JA Scott JD Catterall WA Sather WA. AKAP-anchored PKA maintains neuronal L-type calcium channel activity and NFAT transcriptional signaling. Cell Rep. (2014) 7:1577–88. 10.1016/j.celrep.2014.04.027 24835999 PMC4136445

[173] Sanderson JL Dell'Acqua ML. AKAP signaling complexes in regulation of excitatory synaptic plasticity. Neuroscientist. (2011) 17:321–36. 10.1177/1073858410384740 21498812 PMC3126619

[174] Gilman AG. G proteins: transducers of receptor-generated signals. Annu Rev Biochem. (1987) 56:615–49. 10.1146/annurev.bi.56.070187.003151 3113327

[175] Glovaci I Chapman CA. Activation of phosphatidylinositol-linked dopamine receptors induces a facilitation of glutamate-mediated synaptic transmission in the lateral entorhinal cortex. PLoS ONE. (2015) 10:e0131948. 10.1371/journal.pone.0131948 26133167 PMC4489908

[176] Jones RM Cadby G Melton PE Abraham LJ Whitehouse AJ Moses EK. Genome-wide association study of autistic-like traits in a general population study of young adults. Front Hum Neurosci. (2013) 7:658. 10.3389/fnhum.2013.00658 24133439 PMC3795398

[177] Pagnamenta AT Bacchelli E de Jonge MV Mirza G Scerri TS Minopoli F. Characterization of a family with rare deletions in CNTNAP5 and DOCK4 suggests novel risk loci for autism and dyslexia. Biol Psychiatry. (2010) 68:320–8. 10.1016/j.biopsych.2010.02.002 20346443 PMC2941017

[178] McKenna MC Waagepetersen HS Schousboe A Sonnewald U. Neuronal and astrocytic shuttle mechanisms for cytosolic-mitochondrial transfer of reducing equivalents: current evidence and pharmacological tools. Biochem Pharmacol. (2006) 71:399–407. 10.1016/j.bcp.2005.10.011 16368075

[179] Sica V Kroemer G. IMMP2L: a mitochondrial protease suppressing cellular senescence. Cell Res. (2018) 28:607–8. 10.1038/s41422-018-0051-5 29844577 PMC5993724

[180] Yuan L Zhai L Qian L Huang Ding Y Xiang H. Switching off IMMP2L signaling drives senescence via simultaneous metabolic alteration and blockage of cell death. Cell Res. (2018) 28:625–43. 10.1038/s41422-018-0043-5 29808012 PMC5993829

[181] Adamczyk A Gause CD Sattler R Vidensky S Rothstein JD Singer H. Genetic and functional studies of a missense variant in a glutamate transporter, SLC1A3, in Tourette syndrome. Psychiatr Genet. (2011) 21:90–7. 10.1097/YPG.0b013e328341a307 21233784

[182] Jackson TD Palmer CS Stojanovski D. Mitochondrial diseases caused by dysfunctional mitochondrial protein import. Biochem Soc Trans. (2018) 46:1225–38. 10.1042/BST20180239 30287509

[183] Martensson CU Becker T. Acylglycerol Kinase: mitochondrial protein transport meets lipid biosynthesis. Trends Cell Biol. (2017) 27:700–2. 10.1016/j.tcb.2017.08.006 28867158

[184] Curran SP Leuenberger D Schmidt E Koehler CM. The role of the Tim8p-Tim13p complex in a conserved import pathway for mitochondrial polytopic inner membrane proteins. J Cell Biol. (2002) 158:1017–27. 10.1083/jcb.200205124 12221072 PMC2173223

[185] Kadenbach B. Regulation of mammalian 13-subunit cytochrome c oxidase and binding of other proteins: role of NDUFA4. Trends Endocrinol Metab. (2017) 28:761–70. 10.1016/j.tem.2017.09.003 28988874

[186] Simonic I Nyholt DR Gericke GS Gordon D Matsumoto N Ledbetter DH. Further evidence for linkage of Gilles de la Tourette syndrome (GTS) susceptibility loci on chromosomes 2p11, 8q22 and 11q23-24 in South African Afrikaners. Am J Med Genet. (2001) 105:163–7. 10.1002/ajmg.1192 11304830

[187] Gokhale A Hartwig C Freeman AAH Bassell JL Zlatic SA Sapp Savas C. Systems analysis of the 22q11.2 microdeletion syndrome converges on a mitochondrial interactome necessary for synapse function and behavior. J Neurosci. (2019) 39:3561–81. 10.1523/JNEUROSCI.1983-18.2019 30833507 PMC6495129

[188] Knight S Coon H Johnson M Leppert MF Camp NJ McMahon WM. Linkage analysis of Tourette syndrome in a large Utah pedigree. Am J Med Genet B Neuropsychiatr Genet. (2010) 153B:656–62. 10.1002/ajmg.b.31035 19777563 PMC2923637

[189] Matsumoto N David DE Johnson EW Konecki D Burmester JK Ledbetter DH. Breakpoint sequences of an 1;8 translocation in a family with Gilles de la Tourette syndrome. Eur J Hum Genet. (2000) 8:875–83. 10.1038/sj.ejhg.5200549 11093278

